# Evaluation of potential effects of Plastin 3 overexpression and low-dose SMN-antisense oligonucleotides on putative biomarkers in spinal muscular atrophy mice

**DOI:** 10.1371/journal.pone.0203398

**Published:** 2018-09-06

**Authors:** Eike A. Strathmann, Miriam Peters, Seyyedmohsen Hosseinibarkooie, Frank W. Rigo, C. Frank Bennett, Phillip G. Zaworski, Karen S. Chen, Michael Nothnagel, Brunhilde Wirth

**Affiliations:** 1 Institute of Human Genetics, University of Cologne, Cologne, Germany; 2 Center for Molecular Medicine Cologne, University of Cologne, Cologne, Germany; 3 Endocrine Research Unit, Medical clinic and Outpatient clinic IV, University of Munich, Munich, Germany; 4 IONIS Pharmaceuticals, Carlsbad, California, United States of America; 5 PharmOptima, Portage, Michigan, United States of America; 6 SMA Foundation, New York, New York, United States of America; 7 Department of Statistical Genetics and Bioinformatics, Cologne Centre for Genomics, University of Cologne, Cologne, Germany; 8 Institute for Genetics, University of Cologne, Cologne, Germany; 9 Center for Rare Diseases, University Hospital of Cologne, Cologne, Germany; Iowa State University, UNITED STATES

## Abstract

**Objectives:**

Spinal muscular atrophy (SMA) is a devastating motor neuron disorder caused by homozygous loss of the survival motor neuron 1 (*SMN1*) gene and insufficient functional SMN protein produced by the *SMN2* copy gene. Additional genetic protective modifiers such as Plastin 3 (*PLS3*) can counteract SMA pathology despite insufficient SMN protein. Recently, Spinraza, an SMN antisense oligonucleotide (ASO) that restores full-length *SMN2* transcripts, has been FDA- and EMA-approved for SMA therapy. Hence, the availability of biomarkers allowing a reliable monitoring of disease and therapy progression would be of great importance. Our objectives were (i) to analyse the feasibility of SMN and of six SMA biomarkers identified by the BforSMA study in the Taiwanese SMA mouse model, (ii) to analyse the effect of *PLS3* overexpression on these biomarkers, and (iii) to assess the impact of low-dose SMN-ASO therapy on the level of SMN and the six biomarkers.

**Methods:**

At P10 and P21, the level of SMN and six putative biomarkers were compared among SMA, heterozygous and wild type mice, with or without *PLS3* overexpression, and with or without presymptomatic low-dose SMN-ASO subcutaneous injection. SMN levels were measured in whole blood by ECL immunoassay and of six SMA putative biomarkers, namely Cartilage Oligomeric Matrix Protein (COMP), Dipeptidyl Peptidase 4 (DPP4), Tetranectin (C-type Lectin Family 3 Member B, CLEC3B), Osteopontin (Secreted Phosphoprotein 1, SPP1), Vitronectin (VTN) and Fetuin A (Alpha 2-HS Glycoprotein, AHSG) in plasma.

**Results:**

SMN levels were significantly discernible between SMA, heterozygous and wild type mice. However, no significant differences were measured upon low-dose SMN-ASO treatment compared to untreated animals. Of the six biomarkers, only COMP and DPP4 showed high and SPP1 moderate correlation with the SMA phenotype. *PLS3* overexpression neither influenced the SMN level nor the six biomarkers, supporting the hypothesis that *PLS3* acts as an independent protective modifier.

## Introduction

Spinal muscular atrophy (SMA) is the most common genetic cause of early infant death. SMA is characterised by progressive muscle weakness and atrophy of proximal voluntary muscles due to loss of α-motor neurons in the anterior horns of the spinal cord [1, 2]. Impaired maturation, maintenance and function of neuromuscular junctions (NMJ) are hallmarks of SMA [3, 4]. SMA is caused by functional loss of the survival motor neuron 1 (*SMN1* [MIM: 600354]) gene. Importantly, all patients carry a copy gene, *SMN2* [MIM: 601627], which differs from *SMN1* in a translationally silent nucleotide exchange that disrupts an exonic splicing enhancer and creates a new splicing silencer. This causes the skipping of exon 7 in about 90% of *SMN2* transcripts [5–7]. The SMNΔ7 protein is unstable and rapidly degraded [8]. Only about 10% of *SMN2* transcripts are correctly spliced [9]. Consequently, the severity of SMA, which varies from severe type I to adult type IV, inversely correlates with the copy number of *SMN2* [10, 11]. Approximately 60% of individuals with SMA develop the severe type I form (SMA1 [MIM: 253300]); patients usually carry two *SMN2* copies. These patients are never able to sit or walk, their life expectancy is below two years, while nutritional and respiratory support is necessary. Individuals with the intermediate type II SMA (SMA2 [MIM: 253550]) usually carry three *SMN2* copies, the disease starts after 6 months of age; patients are able to sit, but not to walk. Individuals with mild type III SMA (SMA3 [MIM: 253400]) usually carry three to four *SMN2* copies, disease starts after 18 months of life and patients are able to sit and walk, but often became wheelchair bound with disease progression. Individuals with adult type IV SMA (SMA4 [MIM: 271150]) usually carry four to six *SMN2* copies, onset starts after 30 years of life and affected individuals show only very mild motoric dysfunction [10–12].

In addition to the *SMN2* copy number, there are genetic modifiers found in asymptomatic *SMN1*-deleted individuals in SMA discordant families, such as Plastin 3 (*PLS3*, [MIM: 300131]) and Neurocalcin delta (*NCALD*, [MIM: 606722]) that influence the severity of SMA [13, 14]. PLS3 levels have been found to be upregulated in blood and motor neurons differentiated from induced pluripotent stem cells originated from fibroblasts derived from asymptomatic individuals in comparison to their affected siblings [13, 15]. PLS3 is a Ca^2+^-dependent F-actin-binding and -bundling protein restoring F-actin levels in SMA condition [13, 16]. *PLS3* overexpression is able to rescue axonal growth defects and motor neuron function in a broad range of SMA models including mouse, zebrafish, fly and worm [13, 17–21]. SMN is involved in the process of endocytosis in different SMA models [14, 19, 22, 23]. Overexpression of *PLS3* in an SMA mouse model improves endocytosis at the NMJ site, which is the most affected part in SMA condition [19]. Furthermore, PLS3 improves the NMJ structure and function, connectivity and survival in an intermediate SMA mouse model [19].

Recently, encouraging progress has been made with SMA therapy using either antisense oligonucleotides (ASOs), AAV-based gene therapy or small molecules [24]. Most advanced is the therapy with Spinraza (nusinersen), an ASO that blocks an intronic silencer in *SMN2* transcripts and promotes exon 7 inclusion [25]. This has recently been approved by FDA and EMA for SMA therapy [24]. Recently, two clinical studies, one with Spinraza and another one with AAV9-SMN-cDNA have shown significant improvements in motoric abilities and survival of individuals with SMA1 [26, 27]. Despite this progress in SMA therapy, the studies on reliable biomarkers that would allow an easy monitoring of disease and therapy progression are still scarce.

The phenotypic variability and unsteady disease progression of SMA makes it difficult to estimate treatment outcome and to measure patient improvement. Biomarkers that reliably correlate with the severity of SMA can be beneficial to overcome problems associated with motoric tests and cooperativeness, particularly of small children, to participate in these tests. Thereby, SMA biomarkers are useful to achieve information about disease progression and pharmacodynamics in ongoing pharmacological studies. Several work groups tried to identify biomarkers that correlate with disease severity of SMA [28–37]. The BforSMA project was a pilot study to identify candidate protein, transcript and metabolite biomarkers using an unbiased approach [29, 30]. 27 proteins have subsequently been validated and included into a commercial SMA panel (SMA-MAP) [38]. Of these, 10 have been recently analysed in the severe Δ7-SMA mouse model [32].

Previous studies have shown that overexpression of *PLS3* in the severe Taiwanese SMA mouse model restores the functions of the motor neurons and the NMJ, however, did not show improvement in the rate of survival, due to multi-organ dysfunctions caused by low amount of endogenous SMN protein [18]. Also, in the severe Δ7-SMA mouse model, no improvement in survival or motoric abilities has been found [39]. We recently developed an intermediate Taiwanese SMA mouse model by injecting presymptomatically a suboptimal low-dose SMN-ASO (30 μg), which resembled the phenotype of a type II SMA in patients. This treatment prolonged the survival from 16 days to four weeks (26 ± 9.48 days). Additional *PLS3* overexpression from a transgenic allele prolonged the survival from 26 to more than 250 days in more than 60% of mice [19].

Here we analysed the protein levels of SMN and six biomarkers from the BforSMA biomarker panel (COMP [MIM: 600310], DPP4 [MIM: 102720], SPP1 [MIM: 166490], CLEC3B [MIM: 187520], VTN [MIM: 193190], and AHSG [MIM: 138680]) in plasma of severe and intermediate SMA mice. In addition, we aimed to assess whether *PLS3* overexpression or the low-dose systemically administered SMN-ASO-therapy have any impact on these biomarkers.

## Materials and methods

### Mouse breeding

The Taiwanese SMA mouse model FVB.Cg-Tg (SMN2)2Hung Smn1^tm1Hung^/J (stock number 005058) was obtained from Jackson Laboratory [40]. Both alleles of the murine *Smn* have been knocked out in this severely affected mouse model, while it contains two copies per allele of a human *SMN2* transgene (Smn^KO/KO^; SMN2^tg/tg^). Crossing *Smn*^KO/KO^; SMN2^tg/tg^ mice with *Smn*^KO/WT^ in each F1 generation 50% SMA (*Smn*^KO/KO^; *SMN2*^tg/0^) and 50% HET (*Smn*^KO/WT^; *SMN2*^tg/0^) mice were obtained [41]. We further backcrossed these mice for more than seven generations to produce mice with congenic C57BL/6N background [18]. *PLS3* overexpressing transgenic animals were used to generate SMA-*PLS3*het *(Smn*^KO/KO^; *SMN2*^tg/0^; *PLS3*^tg/0^) and SMA-*PLS3*hom (*Smn*^KO/KO^; *SMN2*^tg/0^; *PLS3*^tg/tg^) mice as well as HET-*PLS3*het (*Smn*^KO/WT^; *SMN2*^tg/0^; *PLS3*^tg/0^) and HET-*PLS3*hom (*Smn*^KO/WT^; *SMN2*^tg/0^; *PLS3*^tg/tg^) mice, all on C57BL/6N background. Neonatal mice were ear tagged, tail cut, and genotyped as previously described [18, 41]. In addition, WT C57BL/6N mice were used as controls.

Mice were maintained at the SPF animal facility of the Institute for Genetics at the University of Cologne, kept under a 12 h light cycle, and given a 2918 and 2919 breeding diet (Harlan). Not more than four mice were kept in a micro isolation chamber (Sentinel-cage-system; each air ventilation system was independent of the laboratory air ventilation) with constant access to water and food. The litter consisted of FS 14 (Sniff) and was replaced once per week. All personnel in the animal facility were professional animal caretakers. A scoring system was used to evaluate the burden of each animal according to specific criteria as listed in S1 Table. Each animal was controlled and evaluated daily. If an animal reached more than 10 points (medium burden), it was controlled two times daily. Animals that reached a score of 20 or more (strong burden) were sacrificed immediately by cervical dislocation (P10) or CO_2_ gassing (P21) followed by immediate cervical dislocation and extraction of whole blood into K_2_EDTA-coated tubes to avoid coagulation. To increase the amount of blood, a cervical massage was performed. All animal procedures were conducted in accordance with European, national and institutional guidelines and protocols and were approved by the responsible government authority: Landesamt für Natur, Umwelt und Verbraucherschutz NRW. (Animal Licence: LANUV NRW under the reference numbers 84–02.05.20.12.120, 84–02.04.2014.A006 and 84–02.04.2015.A378).

### ASO injection

On P2 and P3 six animals of each genotype were injected subcutaneously with 30 μg SMN-ASO, as previously described [19]. SMN-ASO (IONIS Pharmaceuticals [42] was diluted in sterile PBS and the concentration was calculated using photometric density (AD260) (10 mg/mL working solution). The subcutaneous injections with a microliter syringe (Hamilton) were administered as reported [42]. This study included two cohorts of mice to allow endpoint whole blood and plasma at P10 in one cohort and longitudinal assessment out to 21 days in another cohort. Untreated SMA mice were only available for comparison at P10 due to a median survival of about 14 days.

### SMN ECL immunoassay and plasma biomarker protein analysis

100–200 μL whole blood from terminally bled mice at P10 or P21 were collected directly into K_2_EDTA-coated tubes to avoid coagulation. The tubes were mixed gently by inversion. Aliquots of 20–50 μL were collected and stored at -80°C and used for SMN protein analysis as previously described [38].

The SMN ECL immunoassay was carried out by PharmOptima as previously described [43]. Prior to the assay, the samples were vortexed and a 5 μL sample was diluted into 795 μL of dilution buffer (1:160 final dilution). Assay plates were read using an MSD 6000 Imager (Meso Scale Discovery). Data reduction from the SMN ECL Immunoassay was performed using software provided with the MSD 6000 Imager. Whole blood SMN values were reported as pg SMN/mL.

The remaining whole blood fraction was transferred into 1.5 mL micro-centrifuge tubes for plasma processing by centrifugation (800 x g, 10 min. at 4°C). The supernatant (clear layer) was carefully removed, transferred into new 1.5 mL micro-centrifuge tubes and aliquots of 25–50 μL were stored for further analysis at -80°C. The protein measurements of the six plasma biomarkers (COMP, DPP4, SPP1, CLEC3B, VTN, and AHSG, which represent a subset of biomarkers of the SMA-MAP panel available for analysis in mice), were carried out by PharmOptima using commercially available antibodies and calibration reagents and multiplex assay plates manufactured by Meso Scale Discovery as previously described [32, 38]. A total of 126 blood samples were collected and 882 measurements were performed in duplicates. The mean value for each analyte was used. Raw data of the plasma concentrations of SMN and the six plasma proteins at both time points are given in the supplemental data (S2 Table).

### Quantitative real time PCR

Approximately 50–100 μL of blood, brain and muscle was collected from four WT mice and three female and three male heterozygous *PLS3* transgenic mice at P21. Isolation of total RNA from mouse tissues was performed using the Purelink RNA Mini Kit (Thermo Fisher Scientific) and the QIAshredder (Qiagen) kit according to the manufacturer`s instructions. 400ng of RNA was reversely transcribed into cDNA (QuantiTect Reverse Transctiption Kit) according to the manufacturer`s instructions. The quantitative real time PCR was performed using PowerSybrGreen Master Mix (Thermo Fisher Scientific) on a StepOnePlus PCR System (Applied Biosystems). Specific primers for the *PLS3* transgene and endogenous *Pls3* were used as previously described [18]. *Hprt* was used as a housekeeper gene. The 2^-ΔΔC^_T_ method was used to calculate relative changes in gene expression [44].

### Statistics

All statistical assessments were performed using R version 2.3.2 and RStudio version 0.99.49.1. We applied Tukey’s method to exclude outliers of each genotype and biomarker from our raw data sets [45] (see S2 Table). For each genotype at each time point and without or with SMN-ASO treatment, a separate cohort of animals was used, therefore an unpaired experimental design was used. In order to account for potentially non-normally distributed data, we applied nonparametric Kruskal-Wallis (KW) tests as *a priori* tests. Since the concentration of each of the seven proteins was measured in every single animal, the KW-tests were corrected for multiple comparisons using the Bonferroni correction [46]. In cases of significant differences, we subsequently applied *post-hoc* Dunn tests to identify the groups that differ significantly from each other [47]. Dunn tests were corrected for multiple comparisons by use of the Holm correction [48].

For correlation analysis of SMN concentrations with the six putative biomarkers, linear models were applied and the Pearson’s Correlation Coefficient r as well as Spearman’s Correlation Coefficient ρ were calculated.

## Results

### Study design and sample collection

**Fig 1:**
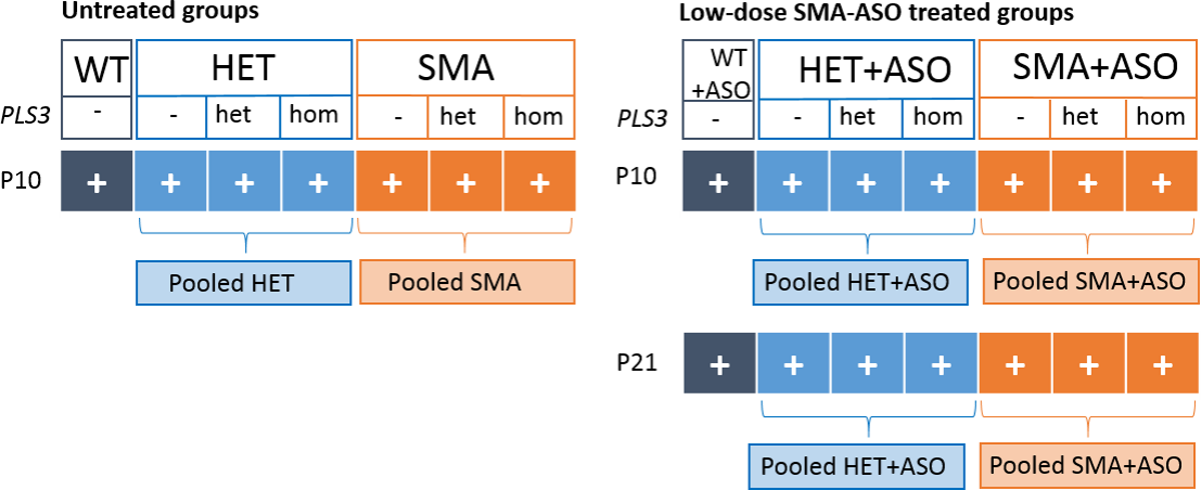
Schematic overview about the mouse genotypes and time points of protein level measurement.

We aimed to address two main questions: First, to assess the impact of *PLS3* overexpression, an SMA protective modifier [13], on SMN protein concentration and on six plasma biomarker analytes (COMP, DPP4, SPP1, CLEC3B, VTN and AHSG). These biomarkers have been identified earlier in SMA-affected individuals by the BforSMA study [30, 38], and verified in the severely affected Δ7SMA mouse model [32]. We investigated the concentration of these analytes in seven different mouse groups: SMA, SMA-*PLS3*het, SMA-*PLS3*hom, HET, HET-*PLS3*het, HET-*PLS3*hom, and WT. Each group consists of three males and three females. Since the mean age of survival of untreated Taiwanese SMA mice is ~16 days and first symptoms occur at P5 [18, 19], the blood samples for all untreated groups have been collected at P10 (7 groups, Fig 1).

Second, modifiers act protectively only in the presence of a certain SMN level, which lies above the SMN amount present in severely affected SMA mice or SMA1-affected individuals [13, 14, 19]. We therefore injected subcutaneously low-dose SMN-ASOs (30 μg at P2 and P3) in the same seven groups (named SMA+ASO, SMA-*PLS3*het+ASO, SMA-*PLS3*hom+ASO, HET+ASO, HET-*PLS3*het+ASO, HET-*PLS3*hom+ASO, and WT+ASO). Since low-dose SMN-ASO-treated SMA mice survive ~26 days [19], we collected blood samples at P10 and P21, which further allowed a longitudinal analysis. Thus, we investigated SMN and the six analytes in all seven ASO-treated groups at two different time points (14 groups; Fig 1).

### SMN blood protein concentration in SMA mice is massively reduced

**Fig 2:**
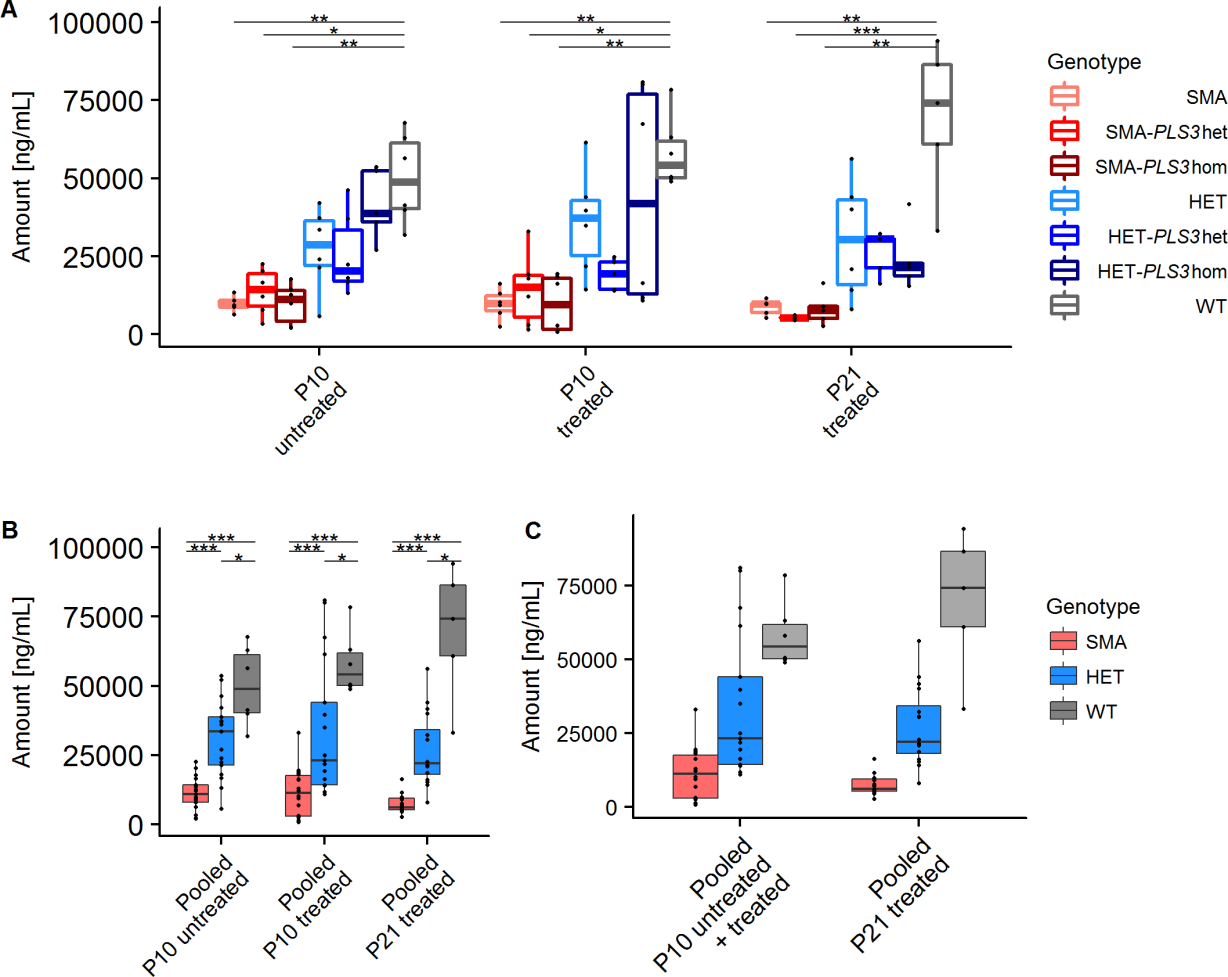
SMN protein concentrations in blood. (A) Multiple comparisons of SMN concentrations in untreated or ASO-treated groups with and without *PLS3* overexpression at P10 and P21 in comparison to WT and WT+ASO, respectively (see also Table 1). (B) Multiple comparisons of pooled groups at P10 and P21 showing SMN concentrations in comparison to WT and WT+ASO, respectively (see also S5 Table). (C) Longitudinal comparisons of pooled ASO-treated groups at P10 and P21 showed no significant differences in the SMN concentration between the two time points (see also S10 Table). (Dunn tests, multiple comparisons, Holm corrected). Only comparisons leading to a significant difference are shown (*P ≤0.05; **P ≤0.01, ***P ≤0.001).

We first verified whether there are significant differences in the concentration of SMN between any two genotypes using the KW-test. Therefore, we compared all genotypes of untreated and ASO-treated samples at P10 as well as ASO-treated samples at P21 (Table 1A). Since significant differences were found between at least two different groups, we next calculated which genotypes differed significantly from each other using *post-hoc* Dunn tests. Based on these results we evaluated the SMN concentration at P10 in each of the three untreated SMA groups (SMA, SMA-*PLS3*het and SMA-*PLS3*hom) and the three untreated HET groups (HET, HET-*PLS3*het and HET-*PLS3*hom) and compared them to untreated WT group. Similarly, the three ASO-treated SMA groups (SMA+ASO, SMA-*PLS3*het+ASO and SMA-*PLS3*hom+ASO) and the three ASO-treated HET groups (HET+ASO, HET-*PLS3*het+ASO and HET-*PLS3*hom+ASO) were compared to WT+ASO animals at P10 and P21. SMN concentrations in all SMA groups, irrespective of *PLS3* overexpression or ASO treatment at both, P10 and P21 but not in HET groups differed significantly from the WT or WT+ASO groups (Table 1B and Fig 2A).

**Table 1:** Comparison of the protein levels of each genotype to the wild type (WT).

A.																	
Treat. group	Comparisons			SMN		COMP		DPP4		SPP1		CLEC3B		VTN		AHSG	
P10 untreated	All against all genotypes			1.33E-03	**	1.10E-04	***	1.45E-04	***	9.51E-02	n.s.	2.05E-01	n.s.	2.64E-04	***	3.83E-03	**
P10 ASO treated	All against all genotypes			9.90E-03	**	1.33E-04	***	3.78E-03	**	3.62E-03	**	2.44E-04	***	3.32E-03	**	7.68E-05	***
P21 ASO treated	All against all genotypes			9.72E-04	***	5.17E-03	**	8.33E-04	***	9.47E-03	**	4.52E-01	n.s.	1.00E+00	n.s.	9.15E-02	n.s.
B.																	
Treat. group	Compared genotypes				SMN		COMP		DPP4		SPP1		CLEC3B		VTN		AHSG	
P10 untreated	SMA	-	WT	2.58E-03	**	3.77E-01	n.s.	3.37E-02	*					3.95E-01	n.s.	6.59E-01	n.s.
	SMA-*PLS3*het	-	WT	1.37E-02	*	1.74E-01	n.s.	8.57E-04	***					7.66E-01	n.s.	8.43E-01	n.s.
	SMA-*PLS3*hom	-	WT	2.02E-03	**	5.51E-02	n.s.	3.47E-05	***					5.18E-01	n.s.	1.00E+00	n.s.
	HET	-	WT	4.66E-01	n.s.	9.06E-01	n.s.	3.13E-01	n.s.					4.82E-01	n.s.	1.86E-01	n.s.
	HET-*PLS3*het	-	WT	3.93E-01	n.s.	1.00E+00	n.s.	9.69E-01	n.s.					1.55E-02	*	8.32E-01	n.s.
	HET-*PLS3*hom	-	WT	9.67E-01	n.s.	6.10E-01	n.s.	1.45E-01	n.s.					1.55E-01	n.s.	1.15E-01	n.s.
P10 ASO treated	SMA+ASO	-	WT+ASO	2.62E-03	**	7.35E-04	***	3.77E-02	*	2.08E-03	**	6.74E-03	**	4.55E-01	n.s.	3.28E-02	*
	SMA-*PLS3*het+ASO	-	WT+ASO	3.93E-02	*	7.75E-04	***	1.06E-04	***	2.41E-03	**	9.46E-04	***	3.97E-02	*	2.06E-01	n.s.
	SMA-*PLS3*hom+ASO	-	WT+ASO	8.09E-03	**	2.37E-03	**	1.00E+00	n.s.	1.00E+00	n.s.	8.67E-05	***	2.31E-03	**	7.12E-01	n.s.
	HET+ASO	-	WT+ASO	1.00E+00	n.s.	7.99E-01	n.s.	4.74E-01	n.s.	1.47E-02	*	4.79E-01	n.s.	5.21E-01	n.s.	4.02E-01	n.s.
	HET-*PLS3*het+ASO	-	WT+ASO	4.00E-01	n.s.	7.97E-01	n.s.	1.55E-01	n.s.	2.65E-01	n.s.	3.90E-01	n.s.	1.00E+00	n.s.	4.46E-01	n.s.
	HET-*PLS3*hom+ASO	-	WT+ASO	9.86E-01	n.s.	7.73E-01	n.s.	6.71E-02	n.s.	1.00E+00	n.s.	6.07E-01	n.s.	4.44E-01	n.s.	7.92E-01	n.s.
P21 ASO treated	SMA+ASO	-	WT+ASO	8.20E-03	**	2.53E-02	*	5.85E-03	**	1.00E+00	n.s.						
	SMA-*PLS3*het+ASO	-	WT+ASO	3.61E-04	***	1.15E-02	*	1.64E-04	***	9.54E-01	n.s.						
	SMA-*PLS3*hom+ASO	-	WT+ASO	2.81E-03	**	3.46E-01	n.s.	8.40E-03	**	7.66E-02	n.s.						
	HET+ASO	-	WT+ASO	4.88E-01	n.s.	1.00E+00	n.s.	5.58E-01	n.s.	1.00E+00	n.s.						
	HET-*PLS3*het+ASO	-	WT+ASO	5.24E-01	n.s.	1.00E+00	n.s.	5.02E-01	n.s.	1.00E+00	n.s.						
	HET-*PLS3*hom+ASO	-	WT+ASO	5.34E-01	n.s.	9.44E-01	n.s.	5.36E-01	n.s.	5.08E-01	n.s.						

*(A) P*-values of *a priori* Kruskal-Wallis tests (Bonferroni corrected for multiple comparisons) and (B) corresponding *post-hoc* Dunn tests (Holm corrected for multiple comparisons) showing that there were significant differences between untreated and ASO-treated groups for SMN and all biomarkers except for five groups (A) and therefore excluded from further analysis (B). Asterisks mark significant differences

(*P ≤0.05;

**P ≤0.01;

***P ≤0.001); n.s. = not significant.

Next, we analysed the influence of *PLS3* overexpression on SMN levels by comparing animals with and without *PLS3* overexpression of the same group (S3 Table). We found no significant differences between animals of the same group and age irrespective of their *PLS3* expression, confirming our former results [18] that PLS3 has no direct influence on the SMN levels.

Similarly, we analysed the influence of low-dose systemically administered SMN-ASO treatment on SMN levels comparing untreated versus ASO-treated animals of the same group and age. In a first step the KW-test showed significant differences between at least two groups (S4A Table). Next, explorative data analysis using *post-hoc* Dunn tests showed no significant differences between untreated and treated animals at P10 (S4B Table), concluding that low-dose SMN-ASO treatment has no measurable influence on SMN levels in blood within any group.

Since PLS3 had no influence on SMN levels and to increase the power of our sample analysis, we pooled untreated SMA, SMA-*PLS3*het and SMA-*PLS3*hom groups (pooled SMA) and SMA+ASO, SMA-*PLS3*het+ASO and SMA-*PLS3*hom+ASO groups (pooled SMA+ASO); similarly, for untreated HET groups (pooled HET) and SMN-ASO-treated HET groups (pooled HET+ASO). Highly significant differences were obtained between pooled SMA or pooled HET and WT at P10 and P21 but also between pooled SMA and pooled HET, clearly allowing discrimination of all three groups (Fig 2B and S5 Table). Thus, pooled SMA+ASO cohort at P10 showed a 5.1-fold and the pooled HET+ASO cohort a 1.7-fold reduction of SMN levels compared to WT+ASO cohort at P10. This difference became even more evident at P21, where pooled SMA+ASO cohort showed a 9.4-fold and pooled HET+ASO a 2.6-fold reduction compared to WT+ASO group (Fig 2B and S6 Table). The differences primarily derive from murine *Smn* copies (2 in WT, 1 in HET and 0 in SMA mice) and to a minor extend from the human *SMN2* transgenes (0 in WT, 2 in HET and SMN mice).

We finally compared the pooled ASO-treated animals at P10 with P21 for longitudinal variation of SMN concentrations (Fig 2C and S10 Table). There were no differences in the SMN concentration between P10 and P21 observed, neither within the pooled SMA+ASO, nor in the pooled HET+ASO or WT+ASO cohort.

Together, these results demonstrate that i) SMN levels in blood are highly significant reduced in pooled SMA mice and still significantly reduced in pooled HET Taiwanese mice compared to WT mice, ii) the fold change reduction of SMN concentrations at P21 is higher than at P10 for pooled SMA and pooled HET groups compared to WT group, iii) low-dose SMN-ASO treatment does not give a measurable elevated SMN level in blood, despite doubling survival of SMA mice, and iv) *PLS3* overexpression has no influence on SMN levels.

### *PLS3* and low dose SMN-ASO treatment have no effect on the concentration of the six biomarkers

Next, we determined the plasma concentration of six putative SMA biomarkers (COMP; DPP4, SPP1, CLEC3B, VTN, and AHSG), which have been identified earlier by the BforSMA study [30, 38]. We followed the same test setup of Bonferroni-corrected KW-tests followed by Holm-corrected Dunn tests as described before. In general, overexpression of *PLS3* had no effect on the concentration of the biomarkers at P10 and P21, except for DPP4 (SMA-*PLS3*het vs. SMA-*PLS3*hom) at P10 (S3 Table). In addition to that, the ASO treatment had also no influence on the concentration of the six putative biomarkers at P10 except for CLEC3B (WT vs. WT+ASO) mice (S4B Table). Similar to SMN, during further analysis we pooled the various groups as indicated in Fig 1.

### COMP, DPP4 and SPP1 are reliable biomarkers for SMA

**Fig 3:**
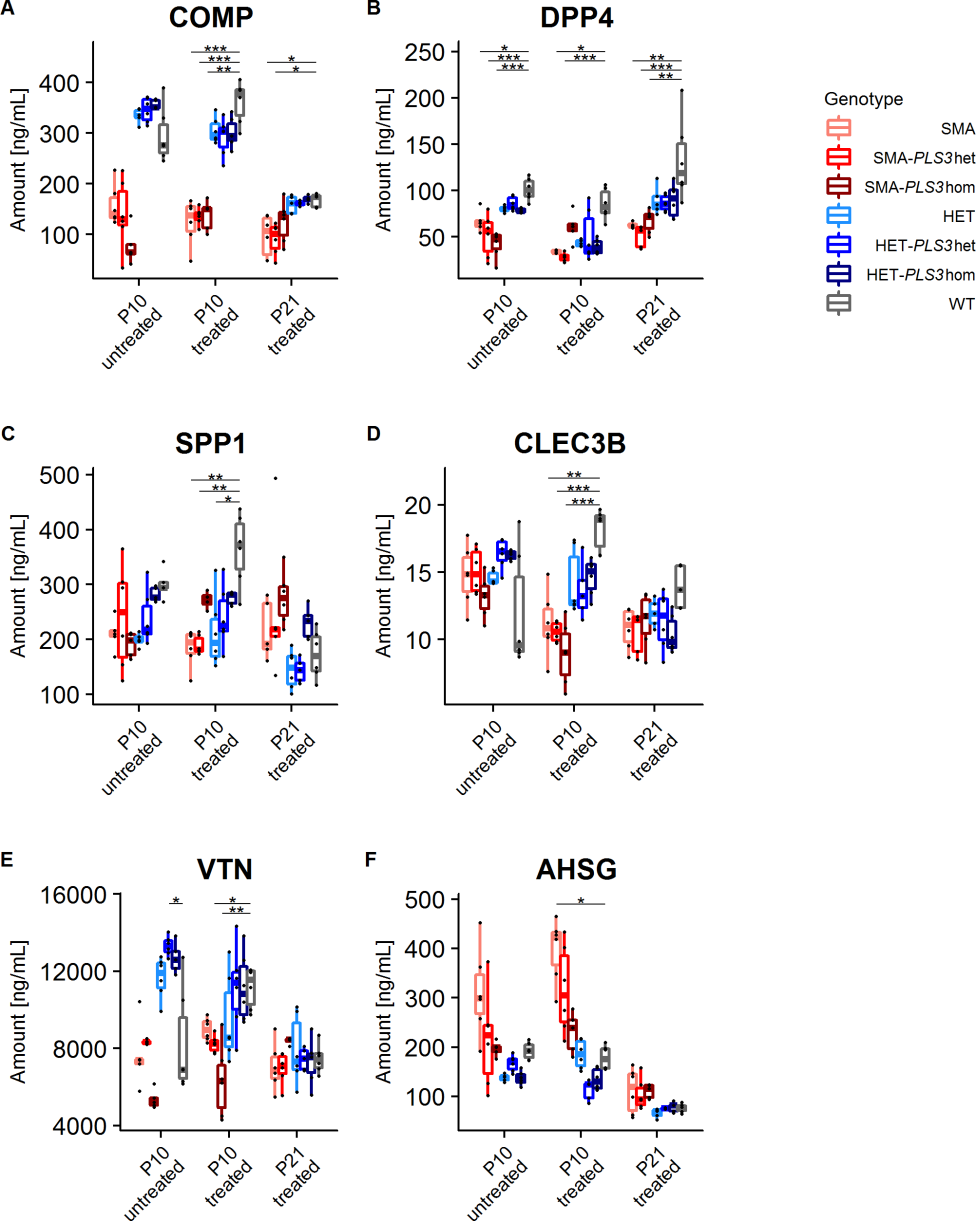
Single group comparison for protein plasma concentrations of six putative biomarkers. Multiple comparisons of untreated and ASO-treated SMA and HET groups with and without *PLS3* overexpression at P10 and P21 showing (A) COMP, (B) DPP4, (C) SPP1, (D) CLEC3B, (E) VTN and (F) AHSG concentrations in comparison to WT and WT+ASO, respectively (see also Table 1). (Dunn tests, Holm-corrected for multiple comparisons). Only comparisons leading to a significant difference are shown (*P ≤0.05; **P ≤0.01, ***P ≤0.001).

**Fig 4:**
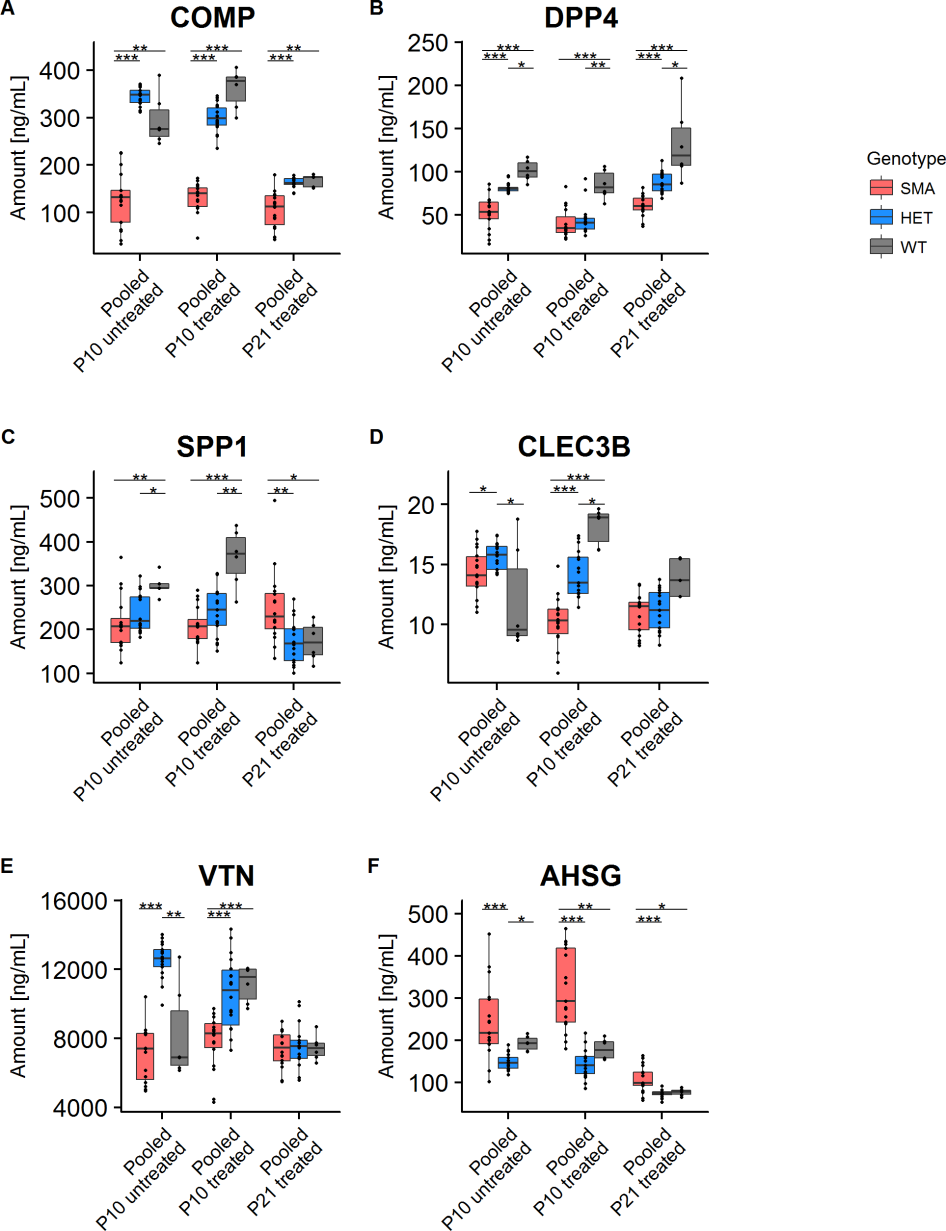
Protein plasma concentrations of six putative biomarkers in pooled groups. Multiple comparisons of unteated and ASO-treated pooled SMA and HET groups at P10 and P21 showing (A) COMP, (B) DPP4, (C) SPP1, (D) CLEC3B, (E) VTN and (F) AHSG concentrations in comparison to WT and WT+ASO, respectively (see also S5 and S6 Tables). (Dunn tests, Holm-corrected for multiple comparisons). Only comparisons leading to a significant difference are shown (*P ≤0.05; **P ≤0.01, ***P ≤0.001).

**COMP** showed a significant difference in untreated pooled SMA group but not in single SMA groups as compared to the WT group. Moreover, COMP was significantly downregulated in all ASO-treated SMA groups except the SMA-*Pls3*hom+ASO group as compared to WT or WT+ASO mice at both time points, P10 and P21 (Figs 3A and 4A and Tables 1B and S5B). COMP was significantly downregulated in pooled SMA+ASO group compared to both, pooled HET+ASO and WT+ASO (Fig 4A and S5B Table). In contrast, pooled HET or pooled HET+ASO mice showed similar concentrations of COMP as WT or WT+ASO groups. Pooled SMA+ASO versus WT+ASO cohort showed a significant decrease of COMP in SMA animals (2.7-fold at P10 and 1.6-fold at P21), while pooled HET or pooled HET+ASO versus WT or WT+ASO showed similar levels (Fig 4A and S5B and S6 Tables). Hence, these data show i) a strong correlation of pooled SMA with reduced COMP levels, ii) a correlation between COMP and very low SMN concentrations as in SMA mice, iii) no influence upon low-dose SMN-ASO treatment, and iv) no influence of *PLS3* overexpression on COMP levels. Moreover, COMP concentration decreased by 2.16-fold from P10 to P21 in WT, which indicates that this marker can be reliably used only in cohorts of the same age.

**DPP4** was significantly downregulated in all single SMA groups except SMA-*PLS3*hom+ASO as compared to WT or WT+ASO groups at P10 and P21 (Fig 3B and Table 1B). Upon pooling the untreated or ASO-treated SMA or HET mice at either P10 or P21, a significant downregulation of DPP4 as compared to WT or WT+ASO was observed (Fig 4B and S5B Table). DPP4 showed very similar reduction of 2.1-fold in pooled SMA at P10 and P21, and 1.9-fold and 1.5-fold in pooled HET mice at P10 and P21, respectively, compared to WT groups (Fig 4B and S6 Table). These data suggest i) a strong correlation of SMA with reduced DPP4 levels, and ii) an association with SMN levels since also HET mice present significantly reduced DPP4 levels.

**SPP1** showed no significant differences between untreated genotypes at P10 (Table 1A), excluding them from further analysis. Of the ASO-treated groups, no differences were found at P21 (Table 1B). Instead, SPP1 was significantly reduced in two of the three SMA groups and in ASO-treated HET mice compared to WT or WT+ASO groups at P10 (Fig 3C and Table 1B). Instead, in untreated or treated pooled SMA or pooled HET groups, SPP1 was significantly downregulated compared to WT or WT+ASO groups (Fig 4C and S5 Table). In WT+ASO mice SPP1 was severely reduced by 2.11-fold from P10 to P21, while the difference between pooled SMA and pooled HET groups even reversed (S5B and S6 Tables). Our data show i) a correlation between SMA and SPP1 ii) a correlation between SMN concentrations and SPP1 especially at P10, and ii) a downregulation of SPP1 between P10 and P21 in WT and pooled HET but not pooled SMA animals.

**CLEC3B** showed high level of variation in WT animals and did not pass the KW-test in untreated mice at P10 and ASO-treated mice at P21 (Table 1A). Further analysis of ASO-treated P10 groups showed that only SMA+ASO but not the HET+ASO groups were significantly different compared to WT+ASO (Fig 3D and Table 1B). Moreover, in pooled cohorts at P10, all groups except SMA vs. WT were significantly different (Fig 4D and S5B Table). We therefore conclude from our data, that CLEC3B is rather not a good and reliable biomarker for SMA.

**VTN** concentration similar to CLEC3B showed high level of variation and did not pass the KW- test at P21 (Table 1A). In SMN-ASO treated groups, only SMA-*PLS3het*+ASO and SMA-*PLS3*hom+ASO at P10 were significantly different compared to WT+ASO (Fig 3E and Table 1B). Overall our data showed quite variable and inconsistent VTN levels and the significant differences were not consistent even among the more powerful pooled groups (Fig 4E and S5 and S6 Tables). Our data does not support that VTN is a reliable and good SMA biomarker in this mouse model.

**AHSG** concentrations, as CLEC3B and VTN, did not show a consistent trend and significance, neither for SMA nor HET groups compared to WT groups (Table 1B). Pooled SMA+ASO groups at both P10 and P21 showed significant differences compared to respective WT+ASO group (Fig 4F and S5 Table). AHSG was the only analyte that showed a higher level in pooled SMA in comparison to WT group at both time points (Fig 4F and S6 Table). As the protein is involved in bone development, the upregulation might be due to delayed development in severely affected SMA mice [49]. Our data suggest that AHSG is a moderately useful SMA biomarker.

In summary, pooled SMA groups showed highly significant decreased levels for three biomarkers, COMP, DPP4 and SPP1, at both time points. In contrast, CLEC3B, VTN and AHSG do not appear as reliable biomarkers in this mouse model based on our data. Low-dose systemically administered SMN-ASO treatment as well as *PLS3* overexpression had in general no influence on these biomarkers.

### Correlations of SMN levels with the concentration of the six biomarkers

**Fig 5:**
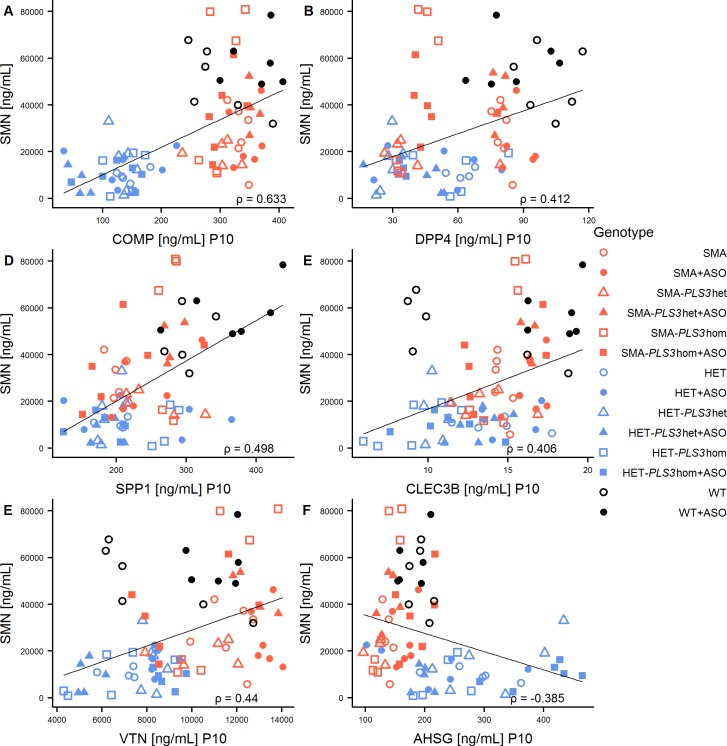
Correlations of SMN plasma protein with the concentration of biomarkers at P10. (A) COMP, (B) DPP4, (C) SPP1, (D) CLEC3B, (E) VTN and (F) AHSG. A linear model is fitted into the plots and the Spearman’s Correlation Coefficient ρ for each linear model is given in S8 Table.

**Fig 6:**
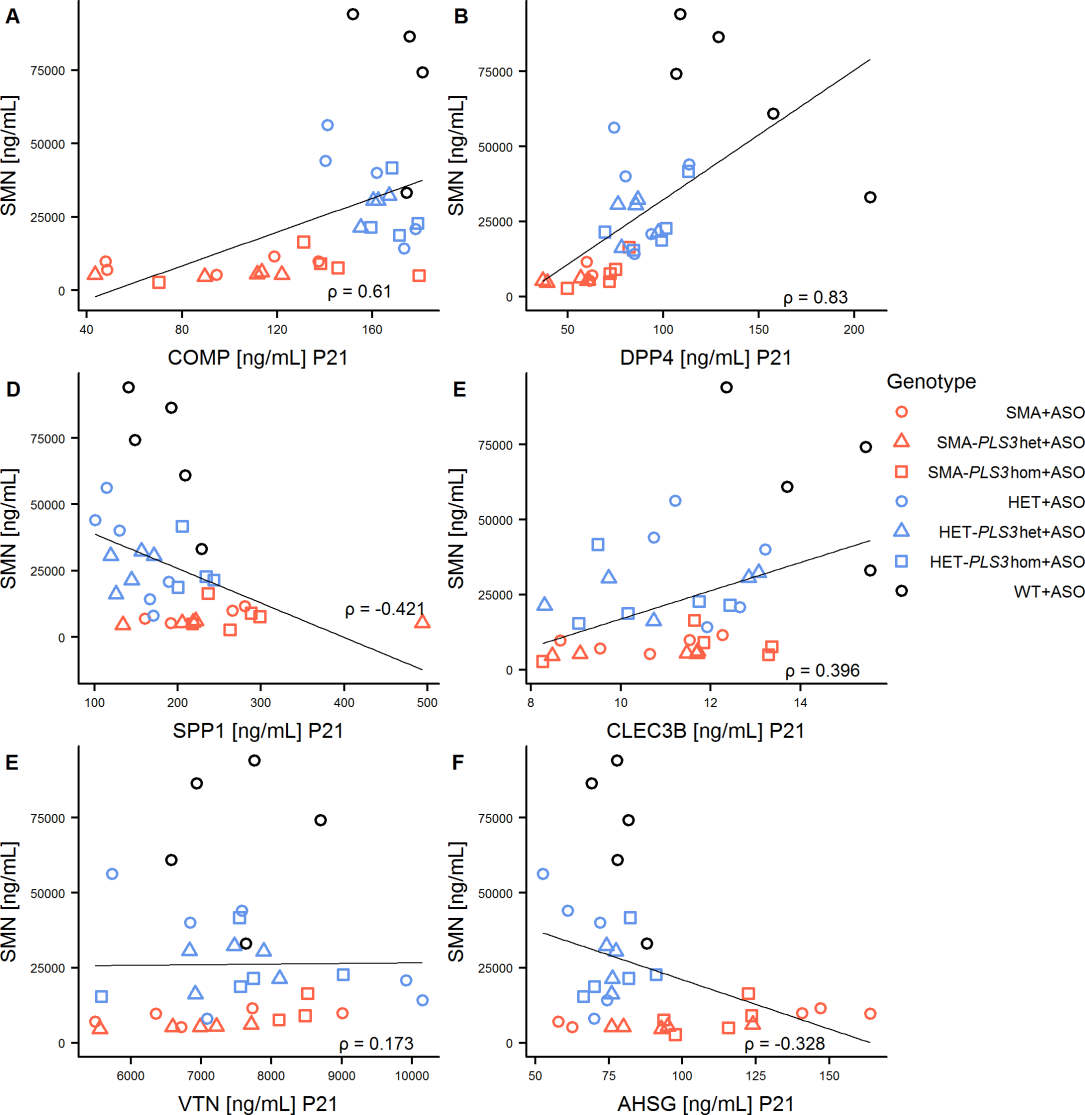
Correlations of SMN plasma protein with the concentration biomarkers at P21. (A) COMP, (B) DPP4, (C) SPP1, (D) CLEC3B, (E) VTN and (F) AHSG. A linear model is fitted into the plots and the Spearman’s Correlation Coefficient ρ for each linear model is given in S8 Table.

Since the SMN levels were massively downregulated in pooled SMA animals at P10, which further increased at P21, and also pooled HET groups showed statistically significant decreased levels compared to WT animals (S5B Table), we correlated the six biomarkers with the SMN concentration and fitted a linear model into the correlation plots (Figs 5 and 6). In addition to that, the *P*-values of the linear models, Pearson’s Correlation Coefficient r as well as Spearman’s Correlation Coefficient ρ were calculated (S8 Table). Our data showed that all linear relations were significant at P10. We found a good correlation (ρ > = 0.5) for COMP and a moderate correlation (ρ > = 0.3) for DPP4, SPP1, CLEC3B and VTN. AHSG was recognized as the only protein with an anti-correlated behaviour with SMN at both P10 and P21. At P21 we found significant linear relations for all plasma proteins except for VTN. COMP and DPP4 were highly correlated to SMN level (ρ > = 0.5) and CLEC3B moderately correlated with SMN (ρ > = 0.3).

### Correlations of protein levels between the six biomarkers

To determine if the biomarkers belong to discrete mechanistic pathways or if they work independently of each other, we used the previously introduced setup to correlate all six putative biomarkers with each other. Spearman correlation coefficients ρ and *P*-values for all comparisons are given in S9 Table. At P10, the strongest correlation is found between COMP, VTN and CLEC3B. Furthermore, good correlations (ρ > = 0.5) were found between COMP and DPP4 as well as between SPP1 and DPP4. AHSG shows at P10 negative Spearman correlation coefficients. It shows weak negative correlations with COMP, VTN and CLEC3B. Interestingly, it shows the strongest positive correlation with SPP1 at P21. Also, a good correlation (ρ > = 0.5) was found between COMP and DPP4 at P21. These data suggest i) that COMP, CLEC3B and VTN may share similar mechanistic pathways, ii) DPP4, SPP1 are only weakly correlated with the other biomarkers and may work independently of each other and iii) AHSG is very weakly anti-correlated with COMP, CLEC3B and VTN and completely independent of DPP4 and SPP1 at P10.

### Comparison of different pooled treatment groups and longitudinal measures

**Fig 7:**
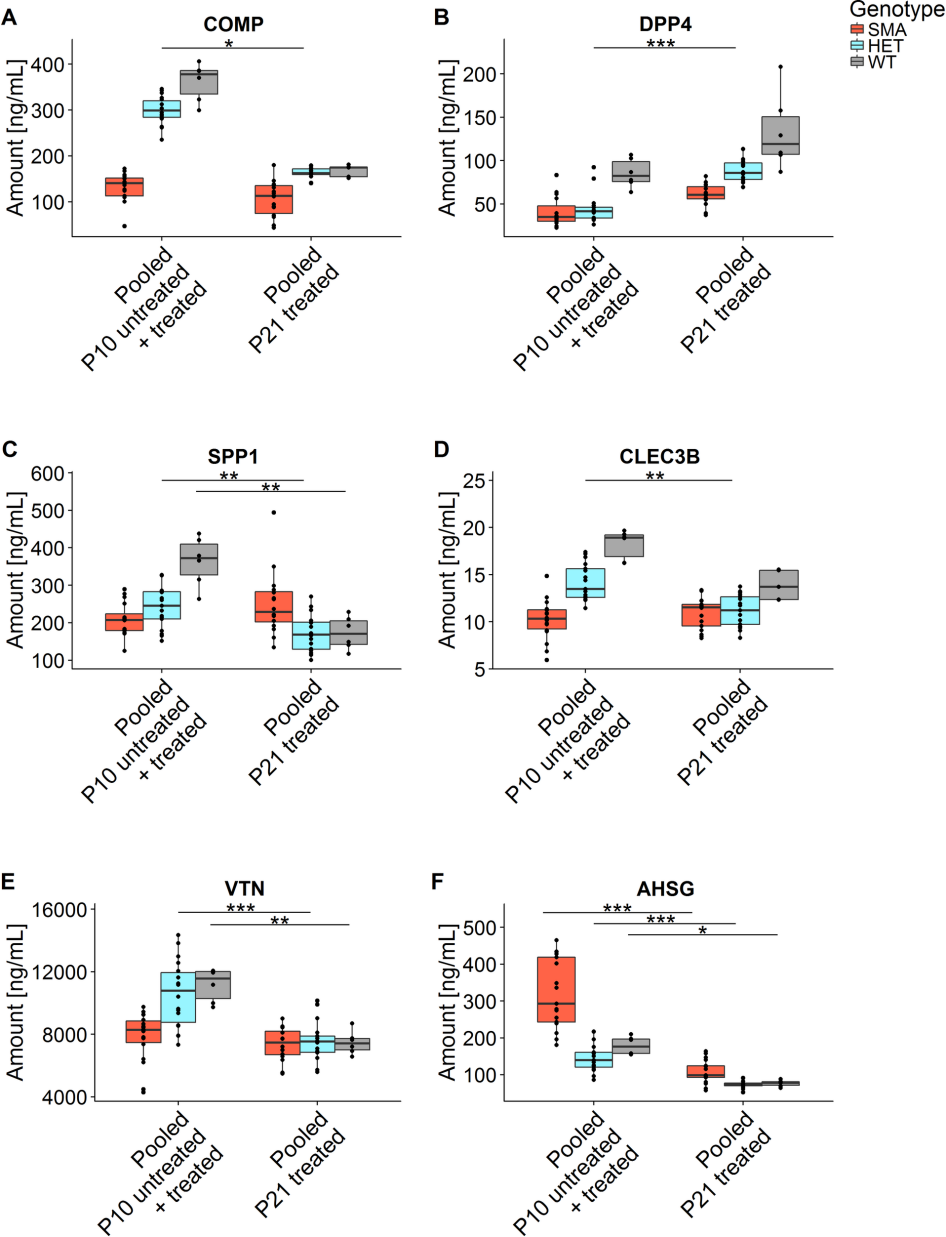
Longitudinal comparisons of P10 with P21 of pooled protein plasma concentrations of six putative biomarkers. Pooled untreated and SMN-ASO treated groups at P10 were longitudinally compared with pooled P21-treated groups for each genotype. Diagrams show (A) COMP, (B) DPP4, (C) SPP1, (D) CLEC3B, (E) VTN and (F) AHSG (see also S10 Table). (Dunn tests, Holm-corrected for multiple comparisons). Only comparisons leading to a significant difference are shown (*P ≤0.05; **P ≤0.01, ***P ≤0.001).

Finally, since *PLS3* overexpression or low-dose SMN-ASO treatment had no effect on SMN or biomarker concentration, we pooled all animals for each of the three genotypes SMA, HET and WT at P10 and those at P21 and compared the concentration of the biomarkers longitudinally (Figs 2C and 7 and S10 Table). The group of all SMA animals showed no significant difference in SMN, COMP, DPP4, SPP1, CLEC3B and VTN but a significant decrease in AHSG between P10 and P21. The group of all HET animals showed no significant difference in SMN, but a significant decrease in COMP, SPP1, CLEC3B, VTN and AHSG and a significant increase in DPP4. The group of all WT animals showed no difference in SMN, COMP, DPP4 and CLEC3B but a significant decrease in SPP1, VTN and AHSG. Thus, only SMN remained constant in all three genotypes between P10 and P21. SPP1, VTN and AHSG showed a natural decrease in HET and WT between P10 and P21, but no change in SMA. DPP4 showed a constant expression in SMA and WT but an increase in HET. CLEC3B showed no difference in WT and SMA but a decrease in HET.

### No sex-specific differences in the concentration of putative biomarkers

No sex-specific differences in the expression of the proteins have been reported in the literature. In order to verify if the sex of the animals may have an effect on the concentration of SMN and the six biomarkers, we again performed Bonferroni-corrected KW-tests followed by Holm-corrected Dunn tests (S11 Table) but did not observe any significant differences in the protein levels of all six biomarkers as well as SMN between female and male mice.

## Discussion

### Usefulness of biomarkers

Current SMA biomarkers will probably not replace the genetic diagnosis by established methods. However, the SMN protein level in most severely affected tissues is not easily measurable. Additionally, motor score tests, such as the modified Hammersmith functional motor scale (MHFMS) can be inappropriate for children under 30 months [50] and may not be comparable to motor score test that are specifically designed for young infants. For these reasons, secure progression and treatment monitoring using blood biomarkers will be of large benefit for SMA patients that are often fragile and immobile. Over the years, several criteria for the evaluation of the predictive value of biomarkers were proposed. Biomarkers should be easily and safely accessible, cost-effective, and significantly associated with the measured value or outcome. According to Wang, biomarkers must show the ability to discriminate affected individuals from those that are not or will not be affected by the disease and must strengthen the correlation agreement between predicted and observed frequency of the outcome according to a risk prediction model [51]. With reference to SMA, biomarkers should be able to discriminate the distinct types of disease severity (SMA type I to IV) and reliably monitor the disease state. Since SMA is a slowly progressing disease, it is important that biomarkers reliably mirror the progression or treatment success at each time point in different unrelated cohorts. Another important aspect is the specificity of biomarkers. An increasing amount of biomarker studies has been published in recent years. Several putative biomarkers were linked to multiple diseases including various cancers and neurodegenerative disorders [52, 53]. Therefore, comorbidity should be taken into account and the usability of a putative biomarker should be critically evaluated with respect to a complex amount of different factors. Hereby, we will discuss our data in scope of this framework and compare it to recently published SMA biomarker studies.

### Effects of sex, *PLS3* overexpression and SMN-ASO treatment on putative SMA biomarkers

**Fig 8:**
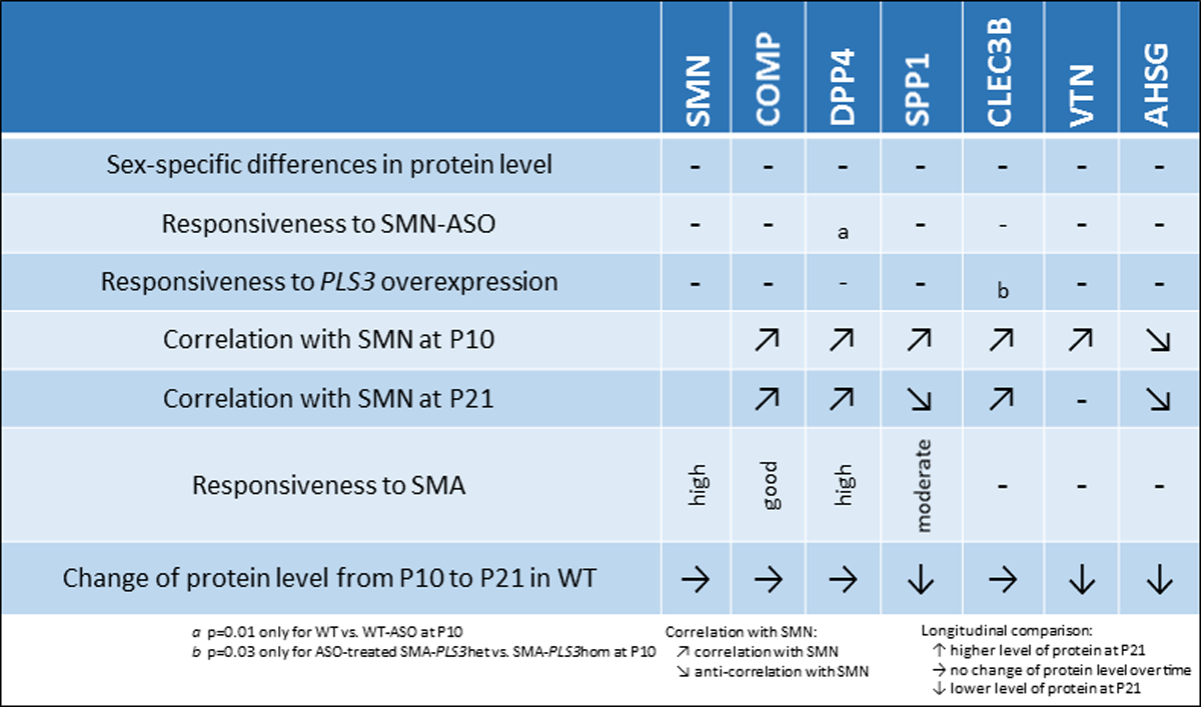
Schematic overview of results for SMN and the six biomarkers.

A schematic summary of the main findings of our study can be found in Fig 8. In line with other biomarker studies, we did not find significant sex-specific differences in the blood concentrations of SMN [29] or the other six putative plasma biomarkers.

Equally, whether *PLS3* overexpression has been achieved genetically from a transgene or from AAV9-expressing *PLS3* cDNA, it led to improved motor neuron axon outgrowth and NMJ functionality, extension of survival and motoric ability especially in low-dose SMN-ASO treated severe SMA mouse models [18, 19, 21, 54]. Nonetheless, *PLS3* overexpression did not influence blood SMN concentration or the six plasma biomarkers tested in this study. This goes well in line with the fact that *PLS3* is normally not expressed in the haematopoietic system but in all solid tissues [55]. *PLS3* expression in blood can be found in only 5% of the control population and in a subset of asymptomatic *SMN1*-deleted individuals, where it acts as a protective modifier [13, 56].

Endogenous *PLS3*/ *Pls3* is an X-linked gene, while the human *PLS3* transgene in our mouse model is inserted in the *Rosa26* locus on chromosome 6 [18]. Endogenous murine *Pls3* is not or at very low levels expressed in blood while no difference in human *PLS3* transgene expression was found among sexes (S1 Fig). Based on our data, *PLS3* might work in other independent pathways, which might not be related to the regulation and release of these six putative biomarkers.

The SMN protein is an obvious choice for the use as a biomarker, as the copy number of *SMN2* is inversely correlated with the disease severity [56]. However, the concentration of SMN in the hematopoietic system may not reflect the abundance in other tissues [57, 58]. The plasma protein concentration, number of transcripts and *SMN2* copy number was analysed as part of the BforSMA study. A correlation between SMN protein levels and disease severity was found. However, SMN and MHFMS were weakly correlated [29].

In former studies we have shown that low-dose SMN-ASO treatment doubles the mean survival in Taiwanese SMA mouse models [19]. Nonetheless, the low-dose subcutaneous SMN-ASO treatment did not affect the concentration of SMN or the other six proteins in the plasma. One explanation might be that the ASOs do not distribute equally *in* vivo. After injection in the blood flow, they accumulate in the liver, kidney, adipocytes, bone marrow and spleen, while haematopoietic cells show a low response to ASOs [59, 60]. Thus, the low-SMN-dose may counteract some of the failures in the development of the liver in severe SMA mouse models and explain the doubling in survival [61]. Additionally, SMN is an intracellular protein and it is highly likely that the origin of the SMN in blood is from erythrocytes, lymphocytes or platelet cells that were lysed during the collection of plasma. Therefore, the amount of SMN in the plasma might not reflect the amount of SMN in the liver or in the motor neurons explaining the increase in survival. Taken together, the treatment with low-dose SMN-ASOs and the *PLS3* overexpression does not modulate the concentration of the analysed plasma proteins.

### Abundancy of the SMA biomarkers over time and comparison to other studies

There are three studies, which have analysed putative SMA plasma biomarkers. In the first study, plasma protein concentrations in 108 children with genetically confirmed SMA have been analysed and the results were correlated with MHFMS scores [30]. In the second study plasma concentrations in a cohort of very young infants (age < six months) have been analysed. They used two different motor function tests for young infants, the Test for Infant Motor Performance Items (TIMPSI) and Children's Hospital of Philadelphia Infant Test of Neuromuscular Disorders (CHOP-INTEND) [31]. In the most recent study, the levels of ten putative biomarkers have been measured at three different time points between SMN-ASO-treated SMAΔ7 and HET mice [32] (summarised in S12, S13 and S14 Tables). There are strong disagreements about the abundancy of putative SMA biomarkers over time between the various studies. A complete overview of the different findings is given in S14 Table. COMP showed a positive correlation with SMN at both time points in our study (S8 Table), while it has been stated as non-responsive to SMN in the SMNΔ7 mouse model [32]. However, COMP has been positively correlated to all three motor scores [30, 31]. DPP4 had a positive correlation with SMN at both time points (Figs 5 and 6 and S8 Table). It has been found to be correlated with MHFMS and TIMPSI [30, 31]. It is not correlated with the age of infants and was very stable over time in the current study [31]. However, in SMNΔ7 mice DPP4 was anti-correlated with SMN at P12, while at later time points there have been no significant correlations with SMN [32]. For SPP1, there are contrary data. It is negatively correlated with the age of infants and with the MHFMS [30, 31]. In the current study, there is a positive correlation with SMN at P10 and a negative correlation at P21. In the SMNΔ7 mice it is the opposite [32]. A correlation of VTN with SMN was found only at P10. In SMNΔ7 mice the results have been also inconsistent with a negative correlation at P30 and positive correlations at P12 and P30 [32]. VTN has been positively correlated with MHFMS [30]. AHSG is the only biomarker in our study with increased concentration in severely affected mice. The concentration was anti-correlated to SMN at both time points. In the SMNΔ7 model, AHSG did not correlate with SMN at any time point [32]. AHSG has been positively correlated with MHFMS and no correlation with age and motor scores in young infants has been found [30, 31]. Interestingly, AHSG is an important non-collagenous protein in bones involves in endochondral ossification [49]. It regulates bone remodelling and calcium metabolism and AHSG-deficient mice showed a shortened femoral bone phenotype [49]. The involvement of AHSG in bone development may explain the increased protein concentration in severely affected SMA animals. For CLEC3B we found a moderate correlation with SMN at both time points. Additionally, it is correlated with TIMPSI [31]. In SMAΔ7 mice CLEC3B has been significantly correlated with SMN only at the earliest time point [32]. One explanation for the strong disagreements between the studies might be that several different model systems and measure values were used. In addition to that, the time points and number of measurement were different between the studies. Taken together, SMN, COMP and DPP4 showed the best performance as SMA biomarkers over time.

### Specificity versus comorbidity

COMP, SPP1 and CLEC3B are associated with human disorders that affect bone and connective tissue. COMP belongs to the Thrombospondin protein family [62] and is mainly located in cartilage, bone tissue and other connective tissues [63–65]. COMP mutations are associated with pseudoachondroplasia [66, 67]. High serum levels are associated with lower risk of myocardial infarction [68]. COMP was suggested as biomarker for disease progression of rheumatoid arthritis, osteoarthritis [69, 70] and liver fibrosis [71], and early cartilage lesions in the knee [72]. Increased serum concentrations were found in patients with rapid hip joint destruction [73]. The serum level of COMP was lower in children with idiopathic scoliosis and high COMP was modestly correlated with high growth velocity [74]. The correlation of COMP with disease severity might be explained by the immobility of severely affected animals. In a recent study, COMP was used as a biomarker for knee osteoarthritis. In that study, 42 women with osteoarthritis in different stages were treated with a well-rounded exercise program over a period of 12 weeks leading to a significant increase of plasma COMP [75]. Paediatric SMA patients are reported to have a low bone mineral density (BMD) and femur fractures are highly prevalent in all SMA subtypes [76, 77]. SPP1 is a non-collagenous matrix protein that helps osteoclasts to migrate and attach to the mineral matrix of bone surfaces [78]. *SPP1* mRNA was downregulated in bone marrow-derived stromal/pre-osteoblast cells of a Taiwanese SMA mouse model compared to the controls [79]. In line with that, we have found that plasma levels of SPP1 were decreased in SMA mice and HET mice at P10. SPP1 is produced by a variety of cell types and is associated not only with bone remodelling but also with immune regulation, inflammation and vascularisation. Thus, expression of *SPP1* is correlated with tumorigenesis, progression and metastasis of several malignancies [80]. Amongst others, misregulation in the level of SPP1 has been reported as possible biomarker for Duchenne Muscular Dystrophy and cardiovascular events [81, 82].

CLEC3B is induced during the mineralisation phase of osteogenesis [83]. It was suggested as a prognostic biomarker for different types of cancer [52, 53].

At P10, COMP, VTN and CLEC3B showed the highest correlation to each other (see S9 Table). According to the Reactome data base (https://reactome.org/), all three proteins are localised in the extracellular region [84]. COMP and VTN interact with several Collagen types and are involved in the extracellular matrix organisation. This may indicate that they are affected by the same mechanistic pathways.

DPP4 and AHSG were both suggested as biomarkers for non-alcoholic fatty liver disease [85, 86]. AHSG is mainly secreted from the liver and inhibits insulin receptor tyrosine kinase [87]. The gene was also suggested as a biomarker for Alzheimer’s disease [88], sepsis [89], cardiovascular mortality and stroke [90, 91].

Taken together, all the putative SMA biomarkers, except for SMN, are linked to several other types of diseases. Additionally, there are differences in the abundancy of plasma concentrations over time. This suggests that a reliable measurement can only be achieved by using a panel of multiple plasma proteins as biomarkers at the same time. This raises the question how many biomarkers are necessary to ensure an accurate measurement. According to a review by Wang the number of necessary biomarkers depends on the degree of correlation between them [51]. If two biomarkers are part of the same pathway, the informative value is very low. Adding more biomarkers from that pathway will not increase the predictive power substantially [51]. However, a combination of several uncorrelated proteins could be used as useful biomarkers for SMA. This suggests that a deeper analysis of the pathways that are related to the putative SMA biomarkers would be beneficial for further biomarker studies. Also, it would be beneficial to analyse more putative biomarkers from the BforSMA pilot study, which identified 97 plasma proteins, 59 plasma metabolites and 44 urine metabolites that correlated with MHFMS scores [29, 30].

### Overall study limitations

The most affected cell type in the SMA condition are motor neurons, which are not accessible for protein measurement in patients. Easy accessible tissues for multiple testing in a patient are blood, saliva or urine. Other accessible tissues but ethically unjustifiable for multiple testing are spinal-cord fluid, muscle or skin biopsy. Measurement of protein concentrations in blood is cost-effectively, safe and easily performable, which is important for treatment monitoring in a clinical setup as well as in biomedical research studies. However, the SMN protein concentration in blood does not necessarily reflect the level of SMN protein in motor neurons. In our study, there were significant differences in the amount of SMN protein between the severely affected SMA mouse groups and the WT, however, the protein levels between HET mice and the WT were largely overlapping. This result is in line with multiple studies, which have shown that SMN mRNA and protein concentrations in blood cannot be used to measure the severity of SMA [29, 34, 92, 93].

One reason for overlap between sample cohorts may be a relatively high degree of mean variance, which we tried to minimise by excluding outliers using Tukey’s method. However, exclusion of data points reduces the power of the statistical analysis. Instead pooling of data can bypass the loss of statistical power. However, this procedure offers some disadvantages: Pooling artificially reduces the observed *P*-values. Additionally, information about individual biological variation can get lost. We performed statistical tests to show that the influence of the *PLS3* transgene on the protein levels in the intermediate mouse model had no effect on the concentrations of the biomarkers, before we pooled the data. Pooling of the samples allowed us to clearly discriminate SMA, HET and WT groups at both P10 and P21.

According to our data, upregulation of PLS3 had no influence on the levels of SMN or the six biomarkers in blood of untreated or SMN-ASO treated animals. While endogenous *Pls3/PLS3* is not expressed in blood of mice and 95% of humans, we tested here the potential impact of the *PLS3* transgene, ubiquitously expressed under the chicken beta promotor [18] and which would resemble the situation in asymptomatic *SMN1*-deleted individuals [13].

One remaining question is about the discordant longitudinal behaviour of the biomarkers in our model system as well as in other systems. This is especially important as we found that the protein level of SMN was stable over time. The intermediate SMN mouse model is an excellent model to study the rescuing effect of *PLS3* overexpression. However, in a clinical scenario, patients are treated with a higher dosage of SMN-ASOs, which could have a stronger impact in the protein levels of biomarkers. Further studies in human study cohorts are needed to evaluate the optimal time windows for biomarker analyses to optimise treatment control.

## Conclusions

In summary, beside SMN, COMP, DPP4 and to some extend SPP1 were identified as the most useful and significantly changed biomarkers in our systemically injected low-dose SMA mouse model. We found significant differences in SMA mice compared to the controls at both time points, P10 and P21. However, all tested plasma proteins were non-responsive to the low-dose SMN-ASO treatment or overexpression of *PLS3* transgene, latter supporting the conclusion that PLS3 acts independent of SMN. There are disagreements about the correlation between the biomarkers and SMN between several studies, which might be explained by the use of different model organisms and the time points used in the various studies. Further studies are needed to evaluate these biomarkers in large cohorts of patients for final clinical application.

## Supporting information

S1 Table Scoring system for the evaluation of animal burden. (DOCX)

S2 Table Raw data of the plasma concentrations of SMN and the six plasma proteins at both time points. Values that were identifies as outliers using the method by Tukey are marked by red shading.
(DOCX)

S3 Table Influence of PLS3 on the levels of SMA putative biomarkers. Holm corrected Dunn tests identified no significant differences except for DPP4 (SMA-*PLS3*het–SMA-*PLS3*hom). The results of the corresponding KW-tests are shown in Table 1A. Asterisks mark significant differences (*P ≤0.05).
(DOCX)

S4 Table Influence of the SMN-ASO on the levels of SMN and the putative biomarkers. (A) *P*-values of *a priori* Kruskal-Wallis tests (Bonferroni corrected for multiple comparisons) and (B) corresponding *post-hoc* Dunn tests (Holm corrected for multiple comparisons) comparing untreated and ASO-treated animals of all seven genotypes at P10 showing that the SMN-ASO treatment has in general no effect on concentrations of SMN and the six biomarkers. Asterisks mark significant differences (*P ≤0.05; **P ≤0.01; ***P ≤0.001).
(DOCX)

S5 Table Comparison of protein levels of pooled genotypes. (A) *P*-values of *a priori* Kruskal-Wallis tests (Bonferroni corrected for multiple comparisons) and (B) corresponding *post-hoc* Dunn tests (Holm corrected for multiple comparisons) comparing untreated and SMN-ASO treated pooled groups at P10 and P21. Asterisks mark significant differences (*P ≤0.05; **P ≤0.01; ***P ≤0.001). Fold changes are given in S6 Table.
(DOCX)

S6 Table Fold change between pooled SMA and pooled HET groups as compared to WT (set to 1) at P10 and P21. (DOCX)

S7 Table Longitudinal comparison of protein levels of SMN-ASO treated genotypes. (A) *P*-values of *a priori* Kruskal-Wallis tests (Bonferroni corrected for multiple comparisons) and (B) corresponding *post-hoc* Dunn tests (Holm corrected for multiple comparisons) for longitudinal comparisons of ASO treated pooled groups. Asterisks mark significant differences (*P ≤0.05; **P ≤0.01; ***P ≤0.001).
(DOCX)

S8 Table Regression and correlation analysis. Correlation of whole blood SMN and plasma analyte levels. *P*-values from a linear model, Pearson’s Correlation Coefficient r and Spearman’s Correlation Coefficient ρ.
(DOCX)

S9 Table Regression and correlation analysis between each of the six biomarkers. Correlation between each biomarker with all other biomarkers. *P*-values from a linear model and Spearman’s Correlation Coefficient ρ.
(DOCX)

S10 Table Longitudinal comparison of protein levels of genotypes. (A) *P*-values of *a priori* Kruskal-Wallis tests (Bonferroni corrected for multiple comparisons) and (B) corresponding *post-hoc* Dunn tests (Holm corrected for multiple comparisons) for longitudinal comparisons of pooled groups. Untreated and SMN-ASO treated genotypes were pooled at P10. Asterisks mark significant differences (*P ≤0.05; **P ≤0.01; ***P ≤0.001).
(DOCX)

S11 Table Comparison of the protein levels between female and male mice of the same genotype. (A) *P*-values of *a priori* Kruskal-Wallis tests (Bonferroni corrected for multiple comparisons) and (B) corresponding *post-hoc* Dunn tests (Holm corrected for multiple comparisons) comparing the concentration of SMN and the six putative biomarkers between male and female animals of each genotype and treatment group showing that there were no sex-specific differences at all. Asterisks mark significant differences (*P ≤0.05; **P ≤0.01; ***P ≤0.001).
(DOCX)

S12 Table Comparison of results from Arnold et al., 2016 and Finkel et al., 2012. (DOCX)

S13 Table Results from Kolb et al., 2016. (DOCX)

S14 Table Comparison of results from Kolb et al., 2016, Arnold et al., 2016, Finkel et al., 2012 and Strathmann et al., 2018. (DOCX)

S1 Fig Expression of *Pls3/PLS3* in different tissues. (A) Endogenous *Pls3* is not expressed in blood but present in brain and muscle tissues. (B) The *PLS3* transgene is expressed in blood of transgenic mice in a low amount. There is no sex-specific difference in the expression between male and female mice (N = 3 for each sex).
(TIF)
