# Supplementary material for: Evaluation of potential effects of Plastin 3 overexpression and low-dose SMN-antisense oligonucleotides on putative biomarkers in spinal muscular atrophy mice
Source: PLoS One. 2018 Sep 6;13(9):e0203398. doi: 10.1371/journal.pone.0203398 (PMC6126849; doi:10.1371/journal.pone.0203398)
Supplement: S1 Table — (DOCX) [file pone.0203398.s001.docx]

**S1 Table.**

| **A. Common criteria** | **Points** |
| --- | --- |
| 1. ***Body weight*** |  |
| - Not influenced | 0 |
| - Reduction of body weight < 5% | 1 |
| - Reduction of body weight 5-10% | 5 |
| - Reduction of body weight 11-19% or rather vertebra and pelvic bones palpable (BCS 2) | 10 |
| - Reduction of body weight ≥ 20% or rather vertebra and pelvic bones recognisable (BCS 1) | 20 |
| 1. ***General state of health*** |  |
| - Gleaming, smooth fur; clean body cavities, clear eyes | 0 |
| - Defects of the fur (decreased or increased body care) | 1 |
| - Blunt fur, untended body cavities, dull eyes, increased muscle tonus, minor scratches (no inflammation) | 5 |
| - Dirty fur, clotted/wet body cavities, abnormal posture, dull eyes, high muscle tonus, bent back, leaning head, medium scratches/ skin injuries (medium reddening, swelling, decelerated/defective wound healing) | 10 |
| - Cramps, paralysis, massive scratches/ skin injuries (medium reddening, swelling, defective wound healing) | 20 |
| 1. ***Spontaneous behaviour*** |  |
| - Normal behaviour (e.g.: sleeping, reaction to touching, curiosity, social contact/interaction) | 0 |
| - Minor departure from normal behaviour (e.g.: reduced reaction, normal food and water uptake) | 1 |
| - Extraordinary behaviour (e.g.: reduced or increased motoric abilities) | 5 |
| - Self isolation, lethargy, apathy, distinct hyperkinetic or rather. Stereotypic behaviour, disturbed coordination | 10 |
| - Pain yelling, Auto mutilation/-aggression | 20 |
| ***IV. Clinical indication*** |  |
| - Normal respiration | 0 |
| - Minor departure from normal situation (e.g.: slightly increased respiration) | 1 |
| - Minor palpable temperature departure; clearly increased abdominal respiration | 10 |
| - Strong temperature departure (animal feels hot or cold); respiration sounds, dyspnoea, cyanoses | 20 |
| **B. Specific experimental criteria** |  |
| ***Development of necrosis***   - No necrosis - Necrosis of the tail - Necrosis of the ears - Necrosis of the hind-and forelimbs | 0  1  5  20 |

| **Score** | **Points** |
| --- | --- |
| No burden | 0 |
| Minor burden | 1-9 |
| Medium burden | 10-19 |
| Strong burden | ≥ 20 |
