# Supplementary material for: Evaluation of potential effects of Plastin 3 overexpression and low-dose SMN-antisense oligonucleotides on putative biomarkers in spinal muscular atrophy mice
Source: PLoS One. 2018 Sep 6;13(9):e0203398. doi: 10.1371/journal.pone.0203398 (PMC6126849; doi:10.1371/journal.pone.0203398)
Supplement: S2 Table — Values that were identifies as outliers using the method by Tukey are marked by red shading. (DOCX) [file pone.0203398.s002.docx]

**S2 Table.**

|  | MouseID | Genotype | Sex | SMN (ng/mL) | COMP (ng/mL) | DPP4 (ng/mL) | SPP1 (ng/mL) | CLEC3B (ng/mL) | VTN (ng/mL) | AHSG (ng/mL) |
| --- | --- | --- | --- | --- | --- | --- | --- | --- | --- | --- |
| P10 | p10-164 | SMA | female | 1097 | 226.74 | 86.19 | 251.07 | 16.46 | 10424.89 | 451.98 |
| untreated | p10-182 | SMA | female | 9467 | 132.44 | 65.09 | 205.65 | 14.92 | 7195.61 | 301.77 |
|  | p10-193/3 | SMA | female | 6375 | 147.26 | 128.08 | 613.3 | 17.76 | 14421.79 | 362 |
|  | p10-131 | SMA | male | 10880 | 124.01 | 54.31 | 168.25 | 11.47 | 5784.27 | 191.62 |
|  | p10-132 | SMA | male | 13575 | 180.84 | 67.81 | 216.17 | 14.79 | 7391.64 | 258.23 |
|  | p10-133 | SMA | male | 8866 | 134.7 | 61.04 | 209.83 | 13.14 | 7433.02 | 297.48 |
|  | p10-144 | SMA-*PLS3*het | female | 7951 | 115.96 | 21.42 | 153.73 | 16.72 | 8348.63 | 242.92 |
|  | p10-146 | SMA-*PLS3*het | female | 22657 | 225.76 | 27.76 | 205.86 | 15.72 | 8337.9 | 101.93 |
|  | p10-151 | SMA-*PLS3*het | female | 12200 | 201.54 | 79.98 | 364.79 | 13.51 | 8217.1 | 373.7 |
|  | p10-143 | SMA-*PLS3*het | male | 16652 | 134.66 | 67.29 | 304.44 | 13.38 | 8252.64 | 244.69 |
|  | p10-145 | SMA-*PLS3*het | male | 20407 | 33.7 | 53.52 | 125.04 | 17.11 | 8497.34 | 127.01 |
|  | p10-161 | SMA-*PLS3*het | male | 3467 | 125.67 | 59.69 | 294.07 | 14.02 | 8879.06 | 206.02 |
|  | p10-147 | SMA-*PLS3*hom | female | 17775 | 136.49 | 34.2 | 209.25 | 13.42 | 5437.94 | 283.23 |
|  | p10-153 | SMA-*PLS3*hom | female | 14392 | 41.18 | 16.57 | 87.67 | 15.35 | 5053.22 | 190.43 |
|  | p10-196 | SMA-*PLS3*hom | female | 2207 | 61.46 | 52.21 | 163.26 | 11.02 | 4948.77 | 176.39 |
|  | p10-176 | SMA-*PLS3*hom | male | 9894 | 64.27 | 45.67 | 171.66 | 12.01 | 6167.02 | 199.23 |
|  | p10-200 | SMA-*PLS3*hom | male | 2363 | 79.91 | 53.6 | 211.61 | 13.15 | 5206.1 | 216.69 |
|  | p10-226 | SMA-*PLS3*hom | male | 12724 | 279.36 | 49.98 | 198.1 | 14.15 | 8133.26 | 200.5 |
|  | p10-173 | HET | female | 42157 | 311.73 | 79.33 | 182.47 | 14.33 | 10998.53 | 128.59 |
|  | p10-174 | HET | female | 33626 | 347.34 | 82.06 | 198.56 | 14.22 | 12735.22 | 139.36 |
|  | p10-187 | HET | female | 37344 | 331.27 | 75.25 | 214.31 | 14.29 | 12293.84 | 164.26 |
|  | p10-128 | HET | male | 5766 | 348.63 | 84.97 | 241.33 | 15.12 | 12458.15 | 140.92 |
|  | p10-129 | HET | male | 24038 | 335.64 | 78.1 | 204.03 | 14.18 | 9929.1 | 133.38 |
|  | p10-130 | HET | male | 21501 | 331.01 | 81.08 | 196.43 | 15.32 | 11519.99 | 147.32 |
|  | p10-141 | HET-*PLS3*het | female | 22491 | 370.88 | 82.25 | 272.63 | 15.72 | 13155.17 | 174.49 |
|  | p10-142 | HET-*PLS3*het | female | 37071 | 314.97 | 78.22 | 212.14 | 16.37 | 12646.47 | 145.53 |
|  | p10-159 | HET-*PLS3*het | female | 16757 | 358.08 | 94.34 | 209.42 | 16.75 | 13460.36 | 165.58 |
|  | p10-149 | HET-*PLS3*het | male | 46327 | 369.78 | 86.65 | 322.56 | 17.39 | 13617.66 | 189.07 |
|  | p10-160 | HET-*PLS3*het | male | 13282 | 322.38 | 80.44 | 192.7 | 14.57 | 14040.96 | 152.35 |
|  | p10-172 | HET-*PLS3*het | male | 18125 | 338.95 | 95.43 | 224.2 | 17.44 | 12954.21 | 176.52 |
|  | p10-148 | HET-*PLS3*hom | female | 82404 | 364.33 | 78.13 | 293.43 | 16 | 12597.95 | 132.17 |
|  | p10-181 | HET-*PLS3*hom | female | 36046 | 367.6 | 77.78 | 272.94 | 16.51 | 13840.95 | 118.52 |
|  | p10-194 | HET-*PLS3*hom | female | 27016 | 349.8 | 76.78 | 215.9 | 13.13 | 16886.92 | 127.49 |
|  | p10-150 | HET-*PLS3*hom | male | 38744 | 351.47 | 80.65 | 276.42 | 16.35 | 13025.36 | 158.47 |
|  | p10-152 | HET-*PLS3*hom | male | 52350 | 349.02 | 81.63 | 268.61 | 16.67 | 11825.26 | 146.71 |
|  | p10-178 | HET-*PLS3*hom | male | 53686 | 327.98 | 76.05 | 297.61 | 15.8 | 12152.6 | 138.6 |
|  | p10-202 | WT | female | 32000 | 389.39 | 104.8 | 304.09 | 18.77 | 12723.9 | 207.98 |
|  | p10-203 | WT | female | 39993 | 329.8 | 93.05 | 294.21 | 16.21 | 10499.21 | 173.23 |
|  | p10-204 | WT | female | 56403 | 274.72 | 85.51 | 342.26 | 9.88 | 6913.13 | 174.27 |
|  | p10-205 | WT | male | 62980 | 277.53 | 117.04 | 294.05 | 8.72 | 6164.07 | 192.19 |
|  | p10-206 | WT | male | 67797 | 245.47 | 96.23 | 391.57 | 9.25 | 6298.81 | 193.53 |
|  | p10-218b | WT | male | 41436 | 255.7 | 112.23 | 268.4 | 9.03 | 6900.35 | 215.66 |
|  |  |  |  |  |  |  |  |  |  |  |
|  | MouseID | Genotype | Sex | SMN | COMP | DPP4 | SPP1 | CLEC3B | VTN | AHSG |
| P10 | p10-138 | SMA+ASO | female | 13031 | 150.84 | 35.04 | 183.56 | 11.25 | 8287.45 | 418.62 |
| SMN-ASO treated | p10-140 | SMA+ASO | female | 16258 | 124.16 | 33.13 | 170.88 | 10.4 | 9444.19 | 427.4 |
|  | p10-209 | SMA+ASO | female | 9467 | 100.36 | 36.21 | 212.88 | 10.15 | 8514.9 | 465.35 |
|  | p10-137 | SMA+ASO | male | 10273 | 166.94 | 32.55 | 209.91 | 12.6 | 9748.56 | 434.53 |
|  | p10-139 | SMA+ASO | male | 2531 | 155.71 | 52.82 | 206.29 | 14.85 | 9258.67 | 348.78 |
|  | p10-210 | SMA+ASO | male | 6927 | 46.97 | 21.54 | 124.73 | 7.63 | 8661.2 | 292.55 |
|  | p10-127 | SMA-*PLS3*het+ASO | female | 1492 | 136.12 | 22.51 | 178.34 | 9.83 | 8431.57 | 335.78 |
|  | p10-155 | SMA-*PLS3*het+ASO | female | 12267 | 158.86 | 30.12 | 182.94 | 11.27 | 8920.12 | 212.77 |
|  | p10-220 | SMA-*PLS3*het+ASO | female | 19185 | 143.27 | 34.77 | 214.15 | 11.37 | 8199.59 | 401.68 |
|  | p10-154 | SMA-*PLS3*het+ASO | male | 18044 | 127.04 | 28.01 | 179.56 | 9.73 | 8293.7 | 274.59 |
|  | p10-156 | SMA-*PLS3*het+ASO | male | 3150 | 140.57 | 24.31 | 173.32 | 10.92 | 7738.28 | 242.73 |
|  | p10-219 | SMA-*PLS3*het+ASO | male | 33075 | 110.04 | 29.76 | 207.72 | 10.24 | 7807.5 | 433.8 |
|  | p10-119 | SMA-*PLS3*hom+ASO | female | 2964 | 150.44 | 61.47 | 268.54 | 5.95 | 4295.63 | 277.99 |
|  | p10-243 | SMA-*PLS3*hom+ASO | female | 16316 | 100.12 | 63.62 | 289.67 | 12.08 | 9249.35 | 239.16 |
|  | p10-218a | SMA-*PLS3*hom+ASO | female | 19510 | 151.82 | 83.09 | 371.8 | 10.84 | 7371.25 | 254.95 |
|  | p10-118 | SMA-*PLS3*hom+ASO | male | 898 | 112.76 | 56.3 | 251.11 | 6.85 | 4486.83 | 180.33 |
|  | p10-124 | SMA-*PLS3*hom+ASO | male | 1172 | 25.39 | 13.74 | 70.84 | 8.98 | 6419.94 | 196.2 |
|  | p10-125 | SMA-*PLS3*hom+ASO | male | 18572 | 172.19 | 39.3 | 277 | 9.09 | 6211.1 | 475.59 |
|  | p10-201 | HET+ASO | female | 14356 | 287.25 | 76.13 | 151.78 | 12.86 | 8538.48 | 157.67 |
|  | p10-207 | HET+ASO | female | 34965 | 281.3 | 47.87 | 165.61 | 12.57 | 7917.79 | 175.19 |
|  | p10-208 | HET+ASO | female | 21897 | 290.21 | 42.79 | 178.45 | 12.64 | 8573.79 | 195.98 |
|  | p10-134 | HET+ASO | male | 44059 | 303.09 | 39.91 | 325.76 | 12.29 | 7333.73 | 150.93 |
|  | p10-135 | HET+ASO | male | 61441 | 322.96 | 40.47 | 209.72 | 17.2 | 11631.9 | 217.44 |
|  | p10-136 | HET+ASO | male | 39686 | 345.85 | 46.01 | 245.02 | 17.4 | 12988.8 | 216.5 |
|  | p10-120 | HET-*PLS3*het+ASO | female | 19410 | 235.22 | 26.26 | 218.24 | 11.44 | 7907.43 | 96.67 |
|  | p10-157 | HET-*PLS3*het+ASO | female | 24929 | 312.18 | 33.71 | 232.1 | 14.39 | 11627.81 | 125.59 |
|  | p10-224 | HET-*PLS3*het+ASO | female | 14375 | 337.06 | 79.28 | 327.39 | 16.86 | 12048.92 | 205.72 |
|  | p10-123 | HET-*PLS3*het+ASO | male | 14039 | 303.54 | 41.39 | 282.63 | 12.35 | 9637.36 | 133.49 |
|  | p10-158 | HET-*PLS3*het+ASO | male | 23181 | 302.78 | 32.16 | 214.56 | 13.22 | 11166.7 | 124.52 |
|  | p10-223 | HET-*PLS3*het+ASO | male | 105954 | 261.83 | 92.26 | 169.39 | 22.57 | 14341.56 | 85.97 |
|  | p10-122 | HET-*PLS3*hom+ASO | female | 11776 | 293.95 | 32.36 | 281.75 | 13.48 | 10402.01 | 112.87 |
|  | p10-167 | HET-*PLS3*hom+ASO | female | 67497 | 326.61 | 50.92 | 260.63 | 15.62 | 12568.07 | 158.53 |
|  | p10-244 | HET-*PLS3*hom+ASO | female | 80941 | 342.58 | 41.74 | 284.3 | 16.1 | 13831.07 | 161.2 |
|  | p10-121 | HET-*PLS3*hom+ASO | male | 16446 | 263.59 | 31.61 | 265.68 | 12.6 | 9536.04 | 115.48 |
|  | p10-126 | HET-*PLS3*hom+ASO | male | 10970 | 294.34 | 34.01 | 320.07 | 14.72 | 9355.83 | 120.6 |
|  | p10-166 | HET-*PLS3*hom+ASO | male | 80036 | 282.73 | 45.84 | 285.79 | 15.44 | 11255.69 | 139.68 |
|  | p10-212 | WT+ASO | female | 49991 | 406.21 | 86.77 | 378.36 | 19.27 | 11166.36 | 154.76 |
|  | p10-213 | WT+ASO | female | 50545 | 299.58 | 63.52 | 263.47 | 16.23 | 9988.78 | 157.42 |
|  | p10-214 | WT+ASO | female | 48980 | 370.05 | 75.3 | 366 | 18.85 | 11950.55 | 194.21 |
|  | p10-215 | WT+ASO | male | 63156 | 322.78 | 102.64 | 314.92 | 16.26 | 9736.17 | 157.91 |
|  | p10-216 | WT+ASO | male | 57931 | 384.97 | 106.6 | 420.46 | 18.99 | 12063.37 | 197.22 |
|  | p10-217 | WT+ASO | male | 78452 | 386.23 | 77.51 | 437.9 | 19.66 | 12030.36 | 209.83 |
|  |  |  |  |  |  |  |  |  |  |  |
|  | MouseID | Genotype | Sex | SMN | COMP | DPP4 | SPP1 | CLEC3B | VTN | AHSG |
| P21 | p21-182 | SMA+ASO | female | 11583 | 118.85 | 59.8 | 280.62 | 12.26 | 7729.77 | 147.08 |
| SMN-ASO treated | p21-183 | SMA+ASO | female | 5252 | 94.47 | 61.46 | 191.91 | 10.65 | 6719.95 | 62.53 |
|  | p21-189 | SMA+ASO | female | 9898 | 137.36 | 91.47 | 265.47 | 11.53 | 9007.67 | 140.66 |
|  | p21-184 | SMA+ASO | male | 17596 | 136.38 | 67.73 | 182.28 | 12.23 | 6998.05 | 98.86 |
|  | p21-185 | SMA+ASO | male | 9779 | 47.95 | 26.03 | 480.4 | 8.65 | 6358.44 | 163.86 |
|  | p21-191 | SMA+ASO | male | 7027 | 48.45 | 62.85 | 160.56 | 9.54 | 5491.48 | 57.71 |
|  | p21-156 | SMA-*PLS3*het+ASO | female | 5295 | 43.41 | 36.94 | 493.88 | 9.09 | 6594.35 | 75.91 |
|  | p21-164 | SMA-*PLS3*het+ASO | female | 5316 | 122.03 | 60.32 | 205.32 | 11.69 | 6987.6 | 79.9 |
|  | p21-168 | SMA-*PLS3*het+ASO | female | 6170 | 113.57 | 56.82 | 221.61 | 11.71 | 7710.94 | 123.96 |
|  | p21-155 | SMA-*PLS3*het+ASO | male | 1432 | 66.86 | 86.05 | 1024.01 | 15.88 | 7735.52 | 158.16 |
|  | p21-165 | SMA-*PLS3*het+ASO | male | 4598 | 89.59 | 39.34 | 134.1 | 8.47 | 5560.54 | 92.86 |
|  | p21-169 | SMA-*PLS3*het+ASO | male | 5551 | 111.7 | 60.59 | 218.26 | 11.47 | 7216.87 | 95.08 |
|  | p21-196 | SMA-*PLS3*hom+ASO | female | 5023 | 179.71 | 71.81 | 217.76 | 13.29 | 10416.07 | 115.68 |
|  | p21-221 | SMA-*PLS3*hom+ASO | female | 16446 | 131.18 | 82 | 236.51 | 11.64 | 8513.14 | 122.46 |
|  | p21-222 | SMA-*PLS3*hom+ASO | female | 36014 | 87.78 | 54.86 | 349.86 | 10.05 | 8442.92 | 265.76 |
|  | p21-160 | SMA-*PLS3*hom+ASO | male | 2742 | 70.26 | 49.72 | 262.65 | 8.26 | 6836.45 | 97.59 |
|  | p21-162 | SMA-*PLS3*hom+ASO | male | 9052 | 138.31 | 75 | 288.18 | 11.85 | 8478.1 | 123.52 |
|  | p21-212 | SMA-*PLS3*hom+ASO | male | 7670 | 145.62 | 72.2 | 298.73 | 13.36 | 8106.19 | 93.67 |
|  | p21-152 | HET+ASO | female | 8035 | 246.83 | 160.43 | 170.41 | 17.71 | 7086.2 | 69.99 |
|  | p21-153 | HET+ASO | female | 56288 | 141.3 | 74.37 | 114.36 | 11.21 | 5733.29 | 52.49 |
|  | p21-175 | HET+ASO | female | 44031 | 140.47 | 113.44 | 100.6 | 10.73 | 7580.16 | 61.07 |
|  | p21-149 | HET+ASO | male | 14233 | 173.48 | 84.97 | 166.4 | 11.92 | 10144.71 | 74.34 |
|  | p21-154 | HET+ASO | male | 40087 | 161.97 | 80.35 | 130.27 | 13.22 | 6843.4 | 71.93 |
|  | p21-159 | HET+ASO | male | 20921 | 178.34 | 93.74 | 189.43 | 12.65 | 9910.61 | 95.67 |
|  | p21-150 | HET-*PLS3*het+ASO | female | 30667 | 162.5 | 76.26 | 119.43 | 12.85 | 6834.78 | 57.12 |
|  | p21-163 | HET-*PLS3*het+ASO | female | 50029 | 214.12 | 95.46 | 287.51 | 13.75 | 9840.21 | 89.09 |
|  | p21-178 | HET-*PLS3*het+ASO | female | 16247 | 132.8 | 78.15 | 125.85 | 10.73 | 6917.95 | 75.98 |
|  | p21-151 | HET-*PLS3*het+ASO | male | 32299 | 167.18 | 86.64 | 156.4 | 13.07 | 7472.47 | 74.14 |
|  | p21-157 | HET-*PLS3*het+ASO | male | 30590 | 160.54 | 85.67 | 171.52 | 9.73 | 7889.25 | 77.38 |
|  | p21-179 | HET-*PLS3*het+ASO | male | 21370 | 155.16 | 97.31 | 144.55 | 8.3 | 8116.62 | 76.18 |
|  | p21-167 | HET-*PLS3*hom+ASO | female | 18752 | 171.45 | 99.07 | 200.16 | 10.16 | 7558.34 | 70.05 |
|  | p21-211 | HET-*PLS3*hom+ASO | female | 41725 | 168.39 | 113.15 | 205.45 | 9.49 | 7546.21 | 82.29 |
|  | p21-223 | HET-*PLS3*hom+ASO | female | 192246 | 243.02 | 69.32 | 270.07 | 9.34 | 6480.76 | 191.16 |
|  | p21-158 | HET-*PLS3*hom+ASO | male | 22794 | 179.19 | 101.48 | 234.15 | 11.74 | 9017.47 | 91.01 |
|  | p21-166 | HET-*PLS3*hom+ASO | male | 21479 | 159.45 | 69.39 | 243.44 | 12.43 | 7742.18 | 81.76 |
|  | p21-190 | HET-*PLS3*hom+ASO | male | 15450 | 107.19 | 84.34 | 111.11 | 9.06 | 5581.26 | 66.3 |
|  | p21-207 | WT+ASO | female | 86455 | 175.81 | 129 | 191.94 | 0.08 | 6935.94 | 69.08 |
|  | p21-214 | WT+ASO | female | 33167 | 174.56 | 208.24 | 228.78 | 15.55 | 7637.69 | 87.97 |
|  | p21-215 | WT+ASO | female | 60930 | 107.59 | 157.65 | 208.95 | 13.7 | 6575.61 | 77.96 |
|  | p21-197 | WT+ASO | male | 163364 | 154.29 | 86.97 | 116.89 | 12.32 | 7210.6 | 64.33 |
|  | p21-198 | WT+ASO | male | 74197 | 181.13 | 106.69 | 148.54 | 15.46 | 8696.04 | 81.7 |
|  | p21-208 | WT+ASO | male | 94138 | 151.83 | 109 | 140.62 | 12.35 | 7754.48 | 77.84 |
