# Supplementary material for: Evaluation of potential effects of Plastin 3 overexpression and low-dose SMN-antisense oligonucleotides on putative biomarkers in spinal muscular atrophy mice
Source: PLoS One. 2018 Sep 6;13(9):e0203398. doi: 10.1371/journal.pone.0203398 (PMC6126849; doi:10.1371/journal.pone.0203398)
Supplement: S3 Table — Holm corrected Dunn tests identified no significant differences except for DPP4 (SMA-PLS3het–SMA-PLS3hom). The results of the corresponding KW-tests are shown in Table 1A. Asterisks mark significant differences (*P ≤0.05). (DOCX) [file pone.0203398.s003.docx]

**S3 Table.**

|  | | | | | | | | | | | | | | | | | |
| --- | --- | --- | --- | --- | --- | --- | --- | --- | --- | --- | --- | --- | --- | --- | --- | --- | --- |
|  | | | | | | | | | | | | | | | | | |
| Treat. group | Compared genotypes | | | SMN |  | COMP |  | DPP4 |  | SPP1 |  | CLEC3B |  | VTN |  | AHSG |  |
| P10 | SMA | - | SMA-*PLS3*het | 1.00E+00 |  | 3.56E-01 |  | 7.75E-01 |  |  |  |  |  | 8.83E-01 |  | 4.77E-01 |  |
| untreated | SMA | - | SMA-*PLS3*hom | 4.61E-01 |  | 1.00E+00 |  | 5.42E-01 |  |  |  |  |  | 6.32E-01 |  | 9.16E-01 |  |
|  | SMA-*PLS3*het | - | SMA-*PLS3*hom | 1.00E+00 |  | 1.00E+00 |  | 7.05E-01 |  |  |  |  |  | 4.02E-01 |  | 1.00E+00 |  |
|  | HET | - | HET-*PLS3*het | 9.02E-01 |  | 1.00E+00 |  | 9.00E-01 |  |  |  |  |  | 5.83E-01 |  | 8.30E-01 |  |
|  | HET | - | HET-*PLS3*hom | 7.63E-01 |  | 1.00E+00 |  | 3.41E-01 |  |  |  |  |  | 1.00E+00 |  | 4.46E-01 |  |
|  | HET-*PLS3*het | - | HET-*PLS3*hom | 7.30E-01 |  | 6.65E-01 |  | 7.40E-01 |  |  |  |  |  | 9.88E-01 |  | 8.34E-01 |  |
| P10 | SMA-*PLS3*het | - | SMA | 1.00E+00 |  | 4.90E-01 |  | 1.00E+00 |  | 4.80E-01 |  | 1.00E+00 |  | 9.01E-01 |  | 6.88E-01 |  |
| treated | SMA-*PLS3*hom | - | SMA | 7.54E-01 |  | 1.00E+00 |  | 3.98E-01 |  | 1.83E-01 |  | 8.51E-01 |  | 4.18E-01 |  | 5.21E-01 |  |
|  | SMA-*PLS3*het | - | SMA-*PLS3*hom | 9.45E-01 |  | 1.00E+00 |  | 1.16E-02 | * | 1.93E-01 |  | 1.00E+00 |  | 9.92E-01 |  | 7.73E-01 |  |
|  | HET-*PLS3*het | - | HET | 1.00E+00 |  | 1.00E+00 |  | 6.63E-01 |  | 1.00E+00 |  | 7.90E-01 |  | 7.82E-01 |  | 3.18E-01 |  |
|  | HET-*PLS3*hom | - | HET | 4.33E-01 |  | 1.00E+00 |  | 8.97E-01 |  | 3.03E-01 |  | 4.00E-01 |  | 6.31E-01 |  | 5.81E-01 |  |
|  | HET-*PLS3*het | - | HET-*PLS3*hom | 9.30E-01 |  | 9.62E-01 |  | 3.68E-01 |  | 1.00E+00 |  | 9.17E-01 |  | 1.00E+00 |  | 5.85E-01 |  |
| P21 | SMA-*PLS3*het | - | SMA | 1.00E+00 |  | 1.00E+00 |  | 1.00E+00 |  | 8.87E-01 |  |  |  |  |  |  |  |
| treated | SMA-*PLS3*hom | - | SMA | 1.00E+00 |  | 1.00E+00 |  | 1.00E+00 |  | 8.45E-01 |  |  |  |  |  |  |  |
|  | SMA-*PLS3*het | - | SMA-*PLS3*hom | 1.00E+00 |  | 8.11E-01 |  | 7.51E-01 |  | 1.00E+00 |  |  |  |  |  |  |  |
|  | HET-*PLS3*het | - | HET | 4.83E-01 |  | 4.92E-01 |  | 9.57E-01 |  | 4.98E-01 |  |  |  |  |  |  |  |
|  | HET-*PLS3*hom | - | HET | 9.33E-01 |  | 1.00E+00 |  | 1.00E+00 |  | 7.46E-02 |  |  |  |  |  |  |  |
|  | HET-*PLS3*het | - | HET-*PLS3*hom | 1.00E+00 |  | 1.00E+00 |  | 4.90E-01 |  | 9.63E-02 |  |  |  |  |  |  |  |
