# Supplementary material for: Evaluation of potential effects of Plastin 3 overexpression and low-dose SMN-antisense oligonucleotides on putative biomarkers in spinal muscular atrophy mice
Source: PLoS One. 2018 Sep 6;13(9):e0203398. doi: 10.1371/journal.pone.0203398 (PMC6126849; doi:10.1371/journal.pone.0203398)
Supplement: S4 Table — (A) P-values of a priori Kruskal-Wallis tests (Bonferroni corrected for multiple comparisons) and (B) corresponding post-hoc Dunn tests (Holm corrected for multiple comparisons) comparing untreated and ASO-treated animals of all seven genotypes at P10 showing that the SMN-ASO treatment has in general no effect on concentrations of SMN and the six biomarkers. Asterisks mark significant differences (*P ≤0.05; **P ≤0.01; ***P ≤0.001). (DOCX) [file pone.0203398.s004.docx]

**S4 Table.**

| A. | | | | | | | | | | | | | | | | |
| --- | --- | --- | --- | --- | --- | --- | --- | --- | --- | --- | --- | --- | --- | --- | --- | --- |
| Treatm. group | | Comparisons | SMN |  | COMP |  | DPP4 |  | SPP1 |  | CLEC3B |  | VTN |  | AHSG |  |
| P10 |  | All against all genotypes | 2.46E-05 | *** | 2.03E-08 | *** | 1.10E-06 | *** | 5.45E-04 | *** | 2.46E-05 | *** | 1.24E-06 | *** | 3.73E-07 | *** |
| # |  |  |  |  |  |  |  |  |  |  |  |  |  |  |  |  |
| B. | | | | | | | | | | | | | | | | |
| Compared genotypes | | | SMN |  | COMP |  | DPP4 |  | SPP1 |  | CLEC3B |  | VTN |  | AHSG |  |
| SMA | - | SMA+ASO | 9.56E-01 |  | 1.00E+00 |  | 1.00E+00 |  | 1.00E+00 |  | 1.00E+00 |  | 1.00E+00 |  | 1.00E+00 |  |
| SMA-*PLS3*het | - | SMA-*PLS3*het+ASO | 4.80E-01 |  | 1.00E+00 |  | 1.00E+00 |  | 1.00E+00 |  | 2.69E-01 |  | 1.00E+00 |  | 1.00E+00 |  |
| SMA-*PLS3*hom | - | SMA-*PLS3*hom+ASO | 1.00E+00 |  | 1.00E+00 |  | 1.00E+00 |  | 9.28E-01 |  | 1.00E+00 |  | 1.00E+00 |  | 1.00E+00 |  |
| HET | - | HET+ASO | 1.00E+00 |  | 1.00E+00 |  | 7.82E-01 |  | 1.00E+00 |  | 1.00E+00 |  | 1.00E+00 |  | 1.00E+00 |  |
| HET-*PLS3*het | - | HET-*PLS3*het+ASO | 1.00E+00 |  | 1.00E+00 |  | 3.11E-01 |  | 1.00E+00 |  | 1.00E+00 |  | 1.00E+00 |  | 1.00E+00 |  |
| HET-*PLS3*hom | - | HET-*PLS3*hom+ASO | 1.00E+00 |  | 1.00E+00 |  | 4.27E-01 |  | 1.00E+00 |  | 1.00E+00 |  | 1.00E+00 |  | 9.90E-01 |  |
| WT | - | WT+ASO | 1.00E+00 |  | 1.00E+00 |  | 1.00E+00 |  | 1.00E+00 |  | 3.06E-02 | * | 1.00E+00 |  | 1.00E+00 |  |
