# Supplementary material for: Evaluation of potential effects of Plastin 3 overexpression and low-dose SMN-antisense oligonucleotides on putative biomarkers in spinal muscular atrophy mice
Source: PLoS One. 2018 Sep 6;13(9):e0203398. doi: 10.1371/journal.pone.0203398 (PMC6126849; doi:10.1371/journal.pone.0203398)
Supplement: S5 Table — (A) P-values of a priori Kruskal-Wallis tests (Bonferroni corrected for multiple comparisons) and (B) corresponding post-hoc Dunn tests (Holm corrected for multiple comparisons) comparing untreated and SMN-ASO treated pooled groups at P10 and P21. Asterisks mark significant differences (*P ≤0.05; **P ≤0.01; ***P ≤0.001). Fold changes are given in S6 Table. (DOCX) [file pone.0203398.s005.docx]

**S5 Table.**

| A. | | | | | | | | | | | | | | | | |
| --- | --- | --- | --- | --- | --- | --- | --- | --- | --- | --- | --- | --- | --- | --- | --- | --- |
| Treat. group | | Comparisons | SMN |  | COMP |  | DPP4 |  | SPP1 |  | CLEC3B |  | VTN |  | AHSG |  |
| P10 | | All against all pooled genotypes | 8.09E-08 | *** | 6.81E-12 | *** | 2.87E-08 | *** | 1.78E-03 | ** | 1.45E-07 | *** | 1.91E-08 | *** | 4.64E-08 | *** |
| P21 | | All against all pooled genotypes | 1.29E-05 | *** | 2.33E-04 | *** | 1.48E-05 | *** | 3.86E-02 | * | 9.69E-02 | n.s. | 1.00E+00 | n.s. | 9.54E-03 | ** |
| # | |  |  |  |  |  |  |  |  |  |  |  |  |  |  |  |
| B. | | | | | | | | | | | | | | | | |
|  | Compared groups | | SMN |  | COMP |  | DPP4 |  | SPP1 |  | CLEC3B |  | VTN |  | AHSG |  |
| P10 | Pooled SMA - WT | | 2.16E-05 | *** | 7.54E-03 | ** | 2.88E-06 | *** | 7.95E-03 | ** | 1.89E-01 |  | 2.59E-01 |  | 2.54E-01 |  |
| untreated | Pooled HET - WT | | 4.77E-02 | * | 1.04E-01 |  | 1.68E-02 | * | 4.94E-02 | * | 3.01E-02 | * | 3.62E-03 | ** | 1.84E-02 | * |
|  | Pooled HET – pooled SMA | | 2.18E-04 | *** | 7.88E-08 | *** | 1.96E-04 | *** | 1.17E-01 |  | 4.16E-02 | * | 2.62E-06 | *** | 4.35E-05 | *** |
| P10 | Pooled SMA - WT | | 8.24E-05 | *** | 2.68E-06 | *** | 6.02E-04 | *** | 3.25E-04 | *** | 2.43E-06 | *** | 6.49E-04 | *** | 9.19E-03 | ** |
| treated | Pooled HET - WT | | 4.50E-02 | * | 5.77E-02 |  | 4.74E-03 | ** | 8.42E-03 | ** | 2.53E-02 | * | 1.86E-01 |  | 1.47E-01 |  |
|  | Pooled HET – pooled SMA | | 1.19E-03 | ** | 6.44E-06 | *** | 1.49E-01 |  | 6.79E-02 |  | 8.11E-05 | *** | 5.57E-04 | *** | 6.25E-07 | *** |
| P21 | Pooled SMA - WT | | 8.69E-06 | *** | 1.06E-03 | ** | 5.76E-06 | *** | 3.35E-02 | * |  |  |  |  | 3.33E-02 | * |
| treated | Pooled HET - WT | | 3.26E-02 | * | 3.49E-01 |  | 3.19E-02 | * | 4.53E-01 |  |  |  |  |  | 3.04E-01 |  |
|  | Pooled HET – pooled SMA | | 1.02E-04 | *** | 1.00E-04 | *** | 1.37E-04 | *** | 3.57E-03 | ** |  |  |  |  | 7.10E-04 | *** |
