# Supplementary material for: Evaluation of potential effects of Plastin 3 overexpression and low-dose SMN-antisense oligonucleotides on putative biomarkers in spinal muscular atrophy mice
Source: PLoS One. 2018 Sep 6;13(9):e0203398. doi: 10.1371/journal.pone.0203398 (PMC6126849; doi:10.1371/journal.pone.0203398)
Supplement: S6 Table — (DOCX) [file pone.0203398.s006.docx]

**S6 Table.**

|  |  | **SMN** |  |  | **COMP** |  |  | **DPP4** |  |  | **SPP1** |  |  | **CLEC3B** |  |  | **VTN** |  |  | **AHSG** |  |  |
| --- | --- | --- | --- | --- | --- | --- | --- | --- | --- | --- | --- | --- | --- | --- | --- | --- | --- | --- | --- | --- | --- | --- |
| p10 | SMA | 4.44 | fold | ↓ | 2.32 | fold | ↓ | 1.92 | fold | ↓ | 1.39 | fold | ↓ | 1.20 | fold | ↑ | 1.16 | fold | ↓ | 1.26 | fold | ↑ |
| untreated | HET | 1.62 | fold | ↓ | 1.17 | fold | ↑ | 1.24 | fold | ↓ | 1.25 | fold | ↓ | 1.31 | fold | ↑ | 1.52 | fold | ↑ | 1.30 | fold | ↓ |
|  | WT | 1.00 |  |  | 1.00 |  |  | 1.00 |  |  | 1.00 |  |  | 1.00 |  |  | 1.00 |  |  | 1.00 |  |  |
| p10 | SMA | 5.10 | fold | ↓ | 2.73 | fold | ↓ | 2.10 | fold | ↓ | 1.75 | fold | ↓ | 1.78 | fold | ↓ | 1.42 | fold | ↓ | 0.56 | fold | ↑ |
| treated | HET | 1.71 | fold | ↓ | 1.21 | fold | ↓ | 1.91 | fold | ↓ | 1.51 | fold | ↓ | 1.28 | fold | ↓ | 1.05 | fold | ↓ | 1.22 | fold | ↓ |
|  | WT | 1.00 |  |  | 1.00 |  |  | 1.00 |  |  | 1.00 |  |  | 1.00 |  |  | 1.00 |  |  | 1.00 |  |  |
| p21 | SMA | 9.40 | fold | ↓ | 1.60 | fold | ↓ | 2.19 | fold | ↓ | 1.45 | fold | ↑ | 1.27 | fold | ↓ | 1.02 | fold | ↓ | 1.42 | fold | ↑ |
| treated | HET | 2.57 | fold | ↓ | 1.03 | fold | ↓ | 1.50 | fold | ↓ | 1.01 | fold | ↓ | 1.24 | fold | ↓ | 1.01 | fold | ↑ | 1.04 | fold | ↓ |
|  | WT | 1.00 |  |  | 1.00 |  |  | 1.00 |  |  | 1.00 |  |  | 1.00 |  |  | 1.00 |  |  | 1.00 |  |  |
