# Supplementary material for: Evaluation of potential effects of Plastin 3 overexpression and low-dose SMN-antisense oligonucleotides on putative biomarkers in spinal muscular atrophy mice
Source: PLoS One. 2018 Sep 6;13(9):e0203398. doi: 10.1371/journal.pone.0203398 (PMC6126849; doi:10.1371/journal.pone.0203398)
Supplement: S7 Table — (A) P-values of a priori Kruskal-Wallis tests (Bonferroni corrected for multiple comparisons) and (B) corresponding post-hoc Dunn tests (Holm corrected for multiple comparisons) for longitudinal comparisons of ASO treated pooled groups. Asterisks mark significant differences (*P ≤0.05; **P ≤0.01; ***P ≤0.001). (DOCX) [file pone.0203398.s007.docx]

**S7 Table.**

| A. | | | | | | | | | | | | | | | |
| --- | --- | --- | --- | --- | --- | --- | --- | --- | --- | --- | --- | --- | --- | --- | --- |
| Treatment groups | Comparisons | SMN |  | COMP |  | DPP4 |  | SPP1 |  | CLEC3B |  | VTN |  | AHSG |  |
| P10 vs P21 treated | All against all genotypes | 1.73E-05 | *** | 8.27E-08 | *** | 4.43E-07 | *** | 3.50E-05 | *** | 3.87E-05 | *** | 1.34E-05 | *** | 4.22E-08 | *** |
|  |  |  |  |  |  |  |  |  |  |  |  |  |  |  |  |
| B. | | | | | | | | | | | | | | | |
| P10 | P21 | SMN |  | COMP |  | DPP4 |  | SPP1 |  | CLEC3B |  | VTN |  | AHSG |  |
| SMA+ASO | SMA+ASO | 1.00E+00 |  | 1.00E+00 |  | 1.00E+00 |  | 1.00E+00 |  | 1.00E+00 |  | 5.24E-01 |  | 2.69E-02 | * |
| SMA-*PLS3*het+ASO | SMA-*PLS3*het+ASO | 1.00E+00 |  | 1.00E+00 |  | 1.00E+00 |  | 1.00E+00 |  | 1.00E+00 |  | 1.00E+00 |  | 8.60E-02 |  |
| SMA-*PLS3*hom+ASO | SMA-*PLS3*hom+ASO | 1.00E+00 |  | 1.00E+00 |  | 1.00E+00 |  | 4.79E-01 |  | 1.00E+00 |  | 1.00E+00 |  | 1.00E+00 |  |
| HET+ASO | HET+ASO | 1.00E+00 |  | 1.00E+00 |  | 4.39E-01 |  | 1.00E+00 |  | 1.00E+00 |  | 1.00E+00 |  | 1.61E-02 | * |
| HET-*PLS3*het+ASO | HET-*PLS3*het+ASO | 1.00E+00 |  | 1.00E+00 |  | 4.93E-01 |  | 2.74E-01 |  | 1.00E+00 |  | 1.89E-01 |  | 1.00E+00 |  |
| HET-*PLS3*hom+ASO | HET-*PLS3*hom+ASO | 1.00E+00 |  | 1.00E+00 |  | 6.82E-02 |  | 1.00E+00 |  | 9.78E-02 |  | 6.01E-02 |  | 1.00E+00 |  |
| WT+ASO | WT+ASO | 1.00E+00 |  | 1.00E+00 |  | 1.00E+00 |  | 9.47E-03 | * | 1.00E+00 |  | 6.19E-02 |  | 1.26E-01 |  |
