# Supplementary material for: Evaluation of potential effects of Plastin 3 overexpression and low-dose SMN-antisense oligonucleotides on putative biomarkers in spinal muscular atrophy mice
Source: PLoS One. 2018 Sep 6;13(9):e0203398. doi: 10.1371/journal.pone.0203398 (PMC6126849; doi:10.1371/journal.pone.0203398)
Supplement: S8 Table — Correlation of whole blood SMN and plasma analyte levels. P-values from a linear model, Pearson’s Correlation Coefficient r and Spearman’s Correlation Coefficient ρ. (DOCX) [file pone.0203398.s008.docx]

**S8 Table**.

|  |  | P10 |  |  | P21 |  |
| --- | --- | --- | --- | --- | --- | --- |
|  | *P* | r | ρ | *P* | r | ρ |
| COMP | < 0.001 | 0.605 | 0.633 | 0.004 | 0.490 | 0.610 |
| DPP4 | < 0.001 | 0.399 | 0.412 | < 0.001 | 0.594 | 0.830 |
| SPP1 | < 0.001 | 0.551 | 0.498 | 0.021 | -0.394 | -0.421 |
| CLEC3B | < 0.001 | 0.386 | 0.406 | 0.016 | 0.411 | 0.396 |
| VTN | < 0.001 | 0.424 | 0.440 | 0.956 | 0.010 | 0.173 |
| AHSG | 0.004 | -0.325 | -0.385 | 0.038 | -0.357 | -0.328 |
