# Supplementary material for: Evaluation of potential effects of Plastin 3 overexpression and low-dose SMN-antisense oligonucleotides on putative biomarkers in spinal muscular atrophy mice
Source: PLoS One. 2018 Sep 6;13(9):e0203398. doi: 10.1371/journal.pone.0203398 (PMC6126849; doi:10.1371/journal.pone.0203398)
Supplement: S9 Table — Correlation between each biomarker with all other biomarkers. P-values from a linear model and Spearman’s Correlation Coefficient ρ. (DOCX) [file pone.0203398.s009.docx]

**S9 Table**.

|  | P10 |  |  |  |  | P21 |  |  |  |
| --- | --- | --- | --- | --- | --- | --- | --- | --- | --- |
| Biomarker 1 | Biomarker 2 | rho | P-value |  | Biomarker 1 | Biomarker 2 | rho | P-value |  |
| COMP | VTN | 0.757 | 0.00E+00 | *** | AHSG | SPP1 | 0.753 | 1.05E-07 | *** |
| CLEC3B | VTN | 0.716 | 1.98E-14 | *** | COMP | DPP4 | 0.630 | 1.23E-05 | *** |
| COMP | CLEC3B | 0.623 | 2.56E-10 | *** | COMP | VTN | 0.453 | 2.86E-03 | ** |
| COMP | DPP4 | 0.539 | 1.96E-07 | *** | CLEC3B | VTN | 0.422 | 5.38E-03 | ** |
| DPP4 | SPP1 | 0.503 | 1.55E-06 | *** | COMP | CLEC3B | 0.406 | 7.71E-03 | ** |
| COMP | SPP1 | 0.481 | 4.75E-06 | *** | AHSG | VTN | 0.386 | 1.21E-02 | * |
| DPP4 | CLEC3B | 0.429 | 4.58E-05 | *** | DPP4 | CLEC3B | 0.371 | 1.57E-02 | * |
| DPP4 | VTN | 0.395 | 2.27E-04 | *** | DPP4 | VTN | 0.331 | 3.26E-02 | * |
| SPP1 | CLEC3B | 0.279 | 1.02E-02 | * | SPP1 | VTN | 0.282 | 7.09E-02 |  |
| SPP1 | VTN | 0.257 | 1.86E-02 | * | AHSG | CLEC3B | 0.056 | 7.27E-01 |  |
|  |  |  |  |  | SPP1 | CLEC3B | 0.045 | 7.75E-01 |  |
| AHSG | COMP | -0.498 | 2.01E-06 | *** |  |  |  |  |  |
| AHSG | VTN | -0.403 | 1.64E-04 | *** | AHSG | DPP4 | -0.340 | 2.79E-02 | * |
| AHSG | CLEC3B | -0.311 | 4.03E-03 | ** | DPP4 | SPP1 | -0.315 | 4.28E-02 | * |
| AHSG | DPP4 | -0.147 | 1.83E-01 |  | COMP | SPP1 | -0.202 | 1.98E-01 |  |
| AHSG | SPP1 | -0.072 | 5.14E-01 |  | COMP | AHSG | -0.154 | 3.28E-01 |  |
