# Supplementary material for: Evaluation of potential effects of Plastin 3 overexpression and low-dose SMN-antisense oligonucleotides on putative biomarkers in spinal muscular atrophy mice
Source: PLoS One. 2018 Sep 6;13(9):e0203398. doi: 10.1371/journal.pone.0203398 (PMC6126849; doi:10.1371/journal.pone.0203398)
Supplement: S10 Table — (A) P-values of a priori Kruskal-Wallis tests (Bonferroni corrected for multiple comparisons) and (B) corresponding post-hoc Dunn tests (Holm corrected for multiple comparisons) for longitudinal comparisons of pooled groups. Untreated and SMN-ASO treated genotypes were pooled at P10. Asterisks mark significant differences (*P ≤0.05; **P ≤0.01; ***P ≤0.001). (DOCX) [file pone.0203398.s010.docx]

**S10 Table.**

| A. | | | | | | | | | | | | | | | | |
| --- | --- | --- | --- | --- | --- | --- | --- | --- | --- | --- | --- | --- | --- | --- | --- | --- |
| Time point | Comparisons | | SMN |  | COMP |  | DPP4 |  | SPP1 |  | CLEC3B |  | VTN |  | AHSG |  |
| P10 | All against all pooled genotypes | | 1.81E-08 | *** | 3.00E-11 | *** | 3.18E-09 | *** | 1.81E-04 | *** | 1.64E-07 | *** | 3.11E-06 | *** | 4.18E-11 | *** |
| # | |  |  |  |  |  |  |  |  |  |  |  |  |  |  |  |
| B. | | | | | | | | | | | | | | | | |
| P10 | | P21 | SMN |  | COMP |  | DPP4 |  | SPP1 |  | CLEC3B |  | VTN |  | AHSG |  |
| SMA | | SMA | 4.82E-01 |  | 3.37E-01 |  | 9.35E-02 |  | 3.33E-01 |  | 6.52E-01 |  | 6.04E-01 |  | 4.77E-06 | *** |
| HET | | HET | 7.47E-01 |  | 2.22E-02 | * | 1.98E-05 | *** | 8.36E-03 | ** | 4.66E-03 | ** | 1.43E-04 | *** | 5.67E-04 | *** |
| WT | | WT | 4.18E-01 |  | 1.18E-01 |  | 3.36E-01 |  | 1.49E-03 | ** | 4.77E-01 |  | 6.96E-03 | ** | 1.59E-02 | * |
