# Supplementary material for: Evaluation of potential effects of Plastin 3 overexpression and low-dose SMN-antisense oligonucleotides on putative biomarkers in spinal muscular atrophy mice
Source: PLoS One. 2018 Sep 6;13(9):e0203398. doi: 10.1371/journal.pone.0203398 (PMC6126849; doi:10.1371/journal.pone.0203398)
Supplement: S11 Table — (A) P-values of a priori Kruskal-Wallis tests (Bonferroni corrected for multiple comparisons) and (B) corresponding post-hoc Dunn tests (Holm corrected for multiple comparisons) comparing the concentration of SMN and the six putative biomarkers between male and female animals of each genotype and treatment group showing that there were no sex-specific differences at all. Asterisks mark significant differences (*P ≤0.05; **P ≤0.01; ***P ≤0.001). (DOCX) [file pone.0203398.s011.docx]

**S11 Table.**

| A. | | | | | | | | | | | | | | | | |
| --- | --- | --- | --- | --- | --- | --- | --- | --- | --- | --- | --- | --- | --- | --- | --- | --- |
| Treat. groups | Comparisons |  | SMN |  | COMP |  | DPP4 |  | SPP1 |  | CLEC3B |  | VTN |  | AHSG |  |
| P10 untreated | All against all genotypes (separate sexes) | | 3.65E-02 | * | 9.73E-03 | ** | 1.16E-02 | * | 1.00E+00 | n.s. | 2.28E-01 | n.s. | 1.24E-02 | * | 1.13E-01 | n.s. |
| P10 treated | All against all genotypes (separate sexes) | | 1.84E-01 | n.s. | 8.23E-03 | ** | 1.29E-01 | n.s. | 4.33E-02 | * | 1.54E-02 | * | 6.12E-02 | n.s. | 6.82E-03 | ** |
| P21 treated | All against all genotypes (separate sexes) | | 7.05E-02 | n.s. | 1.29E-01 | n.s. | 5.39E-02 | n.s. | 3.81E-02 | * | 1.00E+00 | n.s. | 1.00E+00 | n.s. | 1.00E+00 | n.s. |
| # |  |  |  |  |  |  |  |  |  |  |  |  |  |  |  |  |
| B. | | | | | | | | | | | | | | | | |
|  | comparisons |  | SMN |  | COMP |  | DPP4 |  | SPP1 |  | CLEC3B |  | VTN |  | AHSG |  |
| P10 untreated | SMA | SMA | 1 |  | 1 |  | 1 |  |  |  |  |  | 1 |  |  |  |
|  | SMA-*PLS3*het | SMA-*PLS3*het | 0.49 |  | 1 |  | 1 |  |  |  |  |  | 1 |  |  |  |
|  | SMA-*PLS3*hom | SMA-*PLS3*hom | 1 |  | 1 |  | 1 |  |  |  |  |  | 1 |  |  |  |
|  | HET | HET | 1 |  | 1 |  | 1 |  |  |  |  |  | 1 |  |  |  |
|  | HET-*PLS3*het | HET-*PLS3*het | 0.97 |  | 1 |  | 1 |  |  |  |  |  | 1 |  |  |  |
|  | HET-*PLS3*hom | HET-*PLS3*hom | 1 |  | 1 |  | 1 |  |  |  |  |  | 1 |  |  |  |
|  | WT | WT | 1 |  | 1 |  | 1 |  |  |  |  |  | 1 |  |  |  |
| P10 treated | SMA | SMA |  |  | 1 |  |  |  | 1 |  | 1 |  |  |  | 1 |  |
|  | SMA-*PLS3*het | SMA-*PLS3*het |  |  | 1 |  |  |  | 1 |  | 1 |  |  |  | 1 |  |
|  | SMA-*PLS3*hom | SMA-*PLS3*hom |  |  | 1 |  |  |  | 1 |  | 1 |  |  |  | 1 |  |
|  | HET | HET |  |  | 1 |  |  |  | 1 |  | 1 |  |  |  | 1 |  |
|  | HET-*PLS3*het | HET-*PLS3*het |  |  | 1 |  |  |  | 1 |  | 1 |  |  |  | 0.96 |  |
|  | HET-*PLS3*hom | HET-*PLS3*hom |  |  | 1 |  |  |  | 1 |  | 1 |  |  |  | 1 |  |
|  | WT | WT |  |  | 1 |  |  |  | 1 |  | 1 |  |  |  | 1 |  |
| P21 treated | SMA | SMA |  |  |  |  |  |  | 1 |  |  |  |  |  |  |  |
|  | SMA-*PLS3*het | SMA-*PLS3*het |  |  |  |  |  |  | 1 |  |  |  |  |  |  |  |
|  | SMA-*PLS3*hom | SMA-*PLS3*hom |  |  |  |  |  |  | 1 |  |  |  |  |  |  |  |
|  | HET | HET |  |  |  |  |  |  | 1 |  |  |  |  |  |  |  |
|  | HET-*PLS3*het | HET-*PLS3*het |  |  |  |  |  |  | 1 |  |  |  |  |  |  |  |
|  | HET-*PLS3*hom | HET-*PLS3*hom |  |  |  |  |  |  | 1 |  |  |  |  |  |  |  |
|  | WT | WT |  |  |  |  |  |  | 1 |  |  |  |  |  |  |  |
