# Supplementary material for: Evaluation of potential effects of Plastin 3 overexpression and low-dose SMN-antisense oligonucleotides on putative biomarkers in spinal muscular atrophy mice
Source: PLoS One. 2018 Sep 6;13(9):e0203398. doi: 10.1371/journal.pone.0203398 (PMC6126849; doi:10.1371/journal.pone.0203398)
Supplement: S12 Table — (DOCX) [file pone.0203398.s012.docx]

**S12 Table.**

|  |  | |  |  |  |  | Finkel et al., 2012 | |
| --- | --- | --- | --- | --- | --- | --- | --- | --- |
|  | Arnold et al., 2016 | | | | | | Correlation of plasma concentration and MHFMS | |
|  | Correlation of whole blood SMN and plasma analyte levels | | | | | |  |  |
|  |  | |  |  |  |  | Human |  |
|  | P12 | | P30 | | P90 | |  |  |
|  | r | *P*-value | r | *P* -value | r | *P* -value | slope | q |
| COMP | not responsive | not responsive | not responsive | not responsive | not responsive | not responsive | 60.78 | 1.29*10^-14^ |
| DPP4 | -0.547 | <0.001 | 0.164 | 0.611 | 0.187 | 0.605 | 65.1 | 4.41*10^-7^ |
| SPP1 | -0.454 | 0.004 | -0.936 | 0.001 | 0.833 | 0.001 | -27.84 | 0.002 |
| CLEC3B | 0.703 | <0.001 | 0.056 | 0.864 | 0.096 | 0.791 | -8.74 | 0.74 |
| VTN | 0.526 | <0.001 | -0.723 | 0.043 | 0.812 | 0.002 | 33.02 | 0.017 |
| AHSG | 0.161 | 0.334 | 0.261 | 0.412 | 0.492 | 0.148 | 56.47 | 1.02*10^-11^ |
