# Supplementary material for: Evaluation of potential effects of Plastin 3 overexpression and low-dose SMN-antisense oligonucleotides on putative biomarkers in spinal muscular atrophy mice
Source: PLoS One. 2018 Sep 6;13(9):e0203398. doi: 10.1371/journal.pone.0203398 (PMC6126849; doi:10.1371/journal.pone.0203398)
Supplement: S13 Table — (DOCX) [file pone.0203398.s013.docx]

**S13 Table.**

| Kolb et al., 2016 | | | | | | | | | | |
| --- | --- | --- | --- | --- | --- | --- | --- | --- | --- | --- |
| Pearson Correlation Coefficients between Baseline Motor Function Test Score and Putative SMA Biomarkers | | | | | | | | | | |
|  | | | | | | | | | | |
| Infants | Age | Age | Age | TIMPSI | TIMPSI | TIMPSI | CHOP-INTEND | CHOP-INTEND | CHOP-INTEND |  |
|  | r | *P* | n | r | *P* | n | r | *P* | n |  |
| COMP | -0.561 | 0.015 | 18 | 0.834 | <0.0001 | 18 | 0.696 | 0.004 | 15 |  |
| DPP4 | 0.029 | 0.909 | 18 | 0.603 | 0.008 | 18 | 0.379 | 0.163 | 15 |  |
| SPP1 | -0.669 | 0.002 | 18 | 0.376 | 0.124 | 18 | 0.1 | 0.724 | 15 |  |
| CLEC3B | -0.132 | 0.602 | 18 | 0.615 | 0.002 | 18 | 0.424 | 0.115 | 15 |  |
| VTN | - | - | - | - | - | - | - | - | - |  |
| AHSG | -0.186 | 0.461 | 18 | 0.268 | 0.282 | 18 | 0.166 | 0.555 | 15 |  |
