# Supplementary material for: Evaluation of potential effects of Plastin 3 overexpression and low-dose SMN-antisense oligonucleotides on putative biomarkers in spinal muscular atrophy mice
Source: PLoS One. 2018 Sep 6;13(9):e0203398. doi: 10.1371/journal.pone.0203398 (PMC6126849; doi:10.1371/journal.pone.0203398)
Supplement: S14 Table — (DOCX) [file pone.0203398.s014.docx]

**S14 Table.**

|  | Arnold et al., 2016 | | | Finkel et al., 2012 | Kolb et al., 2016 | | | Strathmann et al., 2018 | | Overall trend |
| --- | --- | --- | --- | --- | --- | --- | --- | --- | --- | --- |
| Model | SMA∆7 mouse  Correlation with SMN | | | Infants  Correlation with motor score | Infants  Correlation with motor score | | | Taiwanese SMA mouse  Correlation with SMN | |  |
| Time point/ measured variable | P12 | P30 | P90 | MHFMS | Age | TIMPSI | CHOP-INTEND | P10 | P21 |  |
| COMP | n.s. | n.s. | n.s. | ↗ | ↘ | ↗ | ↗ | ↗ | ↗ | ↗ |
| DPP4 | ↘ | n.s. | n.s. | ↗ | n.s. | ↗ | n.s. | ↗ | ↗ | ↗ |
| SPP1 | ↘ | ↘ | ↗ | ↘ | ↘ | n.s. | n.s. | ↗ | ↘ | → |
| CLEC3B | ↗ | ↗ | ↗ | n.s. | n.s. | ↗ | n.s. | ↗ | ↗ | ↗ |
| VTN | ↗ | ↘ | ↗ | ↗ | - | - | - | ↗ | n.s. | ↗ |
| AHSG | n.s. | n.s. | n.s. | ↗ | n.s. | n.s. | n.s. | ↘ | ↘ | → |
| ↗ correlation ↘ anti-correlation → contradicting data n.s. not significant | | | | | | | | | | |
