# Supplementary figures and images for: Evaluation of potential effects of Plastin 3 overexpression and low-dose SMN-antisense oligonucleotides on putative biomarkers in spinal muscular atrophy mice
Source: PLoS One. 2018 Sep 6;13(9):e0203398. doi: 10.1371/journal.pone.0203398 (PMC6126849; doi:10.1371/journal.pone.0203398)

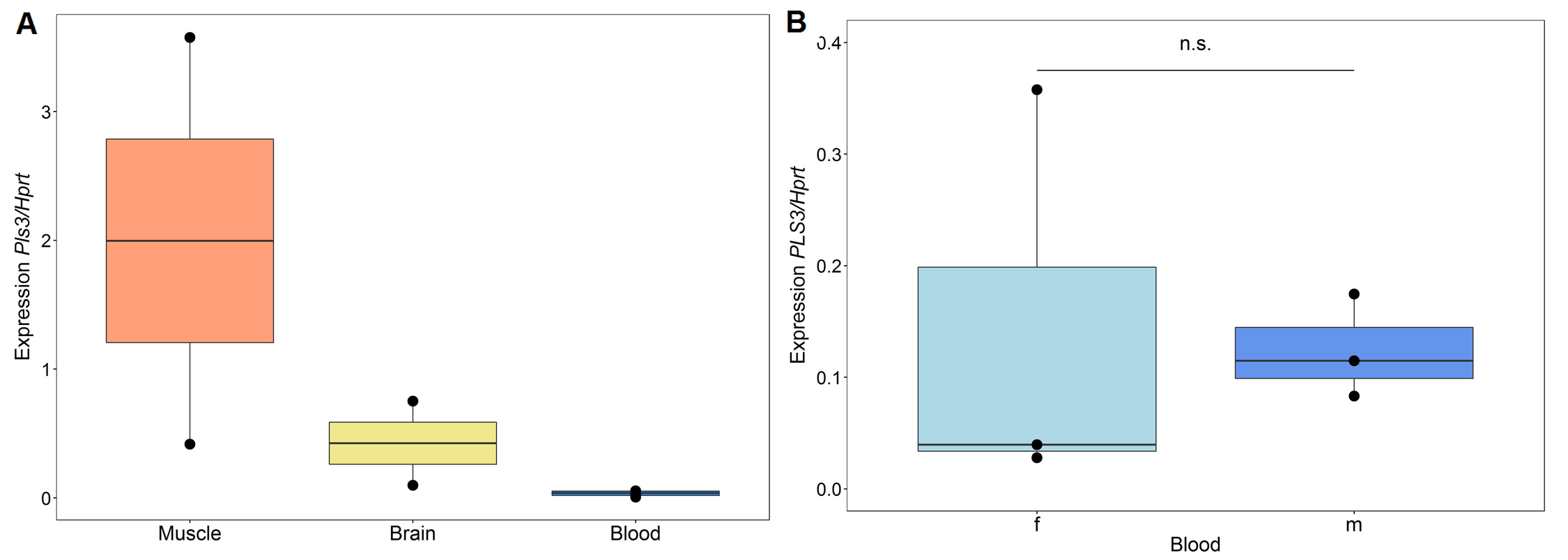

Supplement: S1 Fig — (A) Endogenous Pls3 is not expressed in blood but present in brain and muscle tissues. (B) The PLS3 transgene is expressed in blood of transgenic mice in a low amount. There is no sex-specific difference in the expression between male and female mice (N = 3 for each sex). (TIF) [file pone.0203398.s015.tif]
